# High-throughput discovery of MHC class I- and II-restricted T cell epitopes using synthetic cellular circuits

**DOI:** 10.1038/s41587-024-02248-6

**Published:** 2024-07-02

**Authors:** Ayano C. Kohlgruber, Mohammad H. Dezfulian, Brandon M. Sie, Charlotte I. Wang, Tomasz Kula, Uri Laserson, H. Benjamin Larman, Stephen J. Elledge

**Affiliations:** 1 Division of Genetics, Department of Medicine, Brigham and Women’s Hospital, Boston, MA USA; 2 Department of Genetics, Harvard University Medical School, Boston, MA USA; 3 Division of Immunology, Boston Children’s Hospital, Boston, MA USA; 4 Department of Pathology, Massachusetts General Hospital, Boston, MA USA; 5 Society of Fellows, Harvard University, Cambridge, MA USA; 6 Department of Genetics and Genomic Sciences and Precision Immunology Institute, Icahn School of Medicine at Mount Sinai, New York, NY USA; 7 Institute for Cell Engineering, Division of Immunology, Department of Pathology, Johns Hopkins School of Medicine, Baltimore, MD USA; 8 Howard Hughes Medical Institute, Chevy Chase, MD USA

**Keywords:** Adaptive immunity, Applied immunology

## Abstract

Antigen discovery technologies have largely focused on major histocompatibility complex (MHC) class I-restricted human T cell receptors (TCRs), leaving methods for MHC class II-restricted and mouse TCR reactivities relatively undeveloped. Here we present TCR mapping of antigenic peptides (TCR-MAP), an antigen discovery method that uses a synthetic TCR-stimulated circuit in immortalized T cells to activate sortase-mediated tagging of engineered antigen-presenting cells (APCs) expressing processed peptides on MHCs. Live, tagged APCs can be directly purified for deconvolution by sequencing, enabling TCRs with unknown specificity to be queried against barcoded peptide libraries in a pooled screening context. TCR-MAP accurately captures self-reactivities or viral reactivities with high throughput and sensitivity for both MHC class I-restricted and class II-restricted TCRs. We elucidate problematic cross-reactivities of clinical TCRs targeting the cancer/testis melanoma-associated antigen A3 and discover targets of myocarditis-inciting autoreactive T cells in mice. TCR-MAP has the potential to accelerate T cell antigen discovery efforts in the context of cancer, infectious disease and autoimmunity.

## Abstract (web summary)

T cell-specific antigens are discovered at high throughput without killing target cells.

## Main

CD8^+^ and CD4^+^ T cells recognize peptides presented on cell-surface major histocompatibility complex (MHC) class I and class II molecules, respectively, to survey the intracellular and extracellular landscape for pathogens and assess cellular health^1^. The mechanism by which T cells recognize specific peptide–MHC (pMHC) combinations is through their membrane-bound T cell receptor (TCR). TCR recognition of the cognate pMHC results in T cell activation and induction of various effector functions that are critical for mounting an adaptive immune response.

Knowledge of the antigens that T cells recognize through their TCR is foundational for our understanding of how and why they are involved in the pathology of human diseases such as cancer and autoimmunity. Moreover, harnessing their exquisite specificity holds therapeutic potential and is the basis for successful vaccines and adoptive T cell therapies. However, the inherent diversity of the TCR repertoire, MHC alleles and the peptides that can be presented on any given MHC molecule make the task of mapping T cell antigens a complex problem. In addition, TCRs can be polyspecific, recognizing multiple pMHC combinations, and typically have lower affinity for their antigens (micromolar) compared to antibody–ligand interactions (picomolar to nanomolar)^2,3^. Over the past decade, we and others have developed strategies to determine TCR specificities against tumors, self-antigens, pathogens and allergens^4–14^ but few methods have demonstrated success in assessing TCR reactivities at the proteome scale, hindering high-throughput and unbiased antigen discovery efforts^13,15^. Furthermore, antigen discovery technologies have largely focused on class I-restricted human TCRs and equivalent methods to interrogate class II-restricted CD4^+^ T cells or assess mouse TCR reactivities have not kept pace^5–8^. Ultimately, a universal method that has utility for both human T cell antigen discovery efforts and other frequently used preclinical model organisms, such as mice, and that can be applied for class I-restricted and II-restricted TCRs is highly desirable.

Our lab previously developed T-Scan^13,15^, which can perform genome-wide analysis of TCR specificities across both viral and human proteomes. T-Scan works by using patient or donor T cells programmed with the TCR of interest in a screen against a library that expresses protein fragments presented on MHC molecules in target cells containing a granzyme reporter^13,15^. Upon T cell recognition of target cells expressing the cognate antigen, granzyme B is cytosolically delivered to target cells, which activates the fluorescent reporter for harvesting by cell sorting.

A limitation of this system is the need to obtain fresh primary T cells for each screen. In addition, the assay kills the target cells, limiting the opportunity for further enrichment and subsequent rescreening for signal amplification. Thus, we were motivated to devise an improved screening method that did not rely directly on patient T cells and killing of target cells as part of the recognition assay.

Here we describe a new, cell-based T cell antigen discovery method called TCR mapping of antigenic peptides (TCR-MAP). TCR-MAP enables TCRs with unknown specificity to be queried against a large, peptide tiling library of antigen-presenting cells (APCs) expressing processed peptides on patient-specific or mouse-specific MHC alleles. This system relies on a synthetic circuit expressed in Jurkat cell lines transduced with the TCRs of interest. Upon T cell recognition of the cognate pMHC, the bacterial transpeptidase sortase A (SrtA) is induced and expressed on the cell surface of Jurkats and covalently biotinylates the reciprocal cognate APC. This new method is high throughput and can capture unbiased reactivities against the complete human, mouse or viral proteome or any genetically encoded peptide library of choice. Moreover, it is highly sensitive and can reproducibly discover both high-affinity and low-affinity TCR antigens. We demonstrate the utility of TCR-MAP for antigen discovery efforts for both CD8^+^ and CD4^+^ T cells and for TCRs derived from humans or mice. Application of this technology has the potential to enhance T cell antigen discovery efforts in the context of cancer, infectious disease and autoimmunity.

## Results

### TCR-MAP captures human MHC class I HLA-A2 and class II pMHC–TCR interactions

**Fig. 1:**
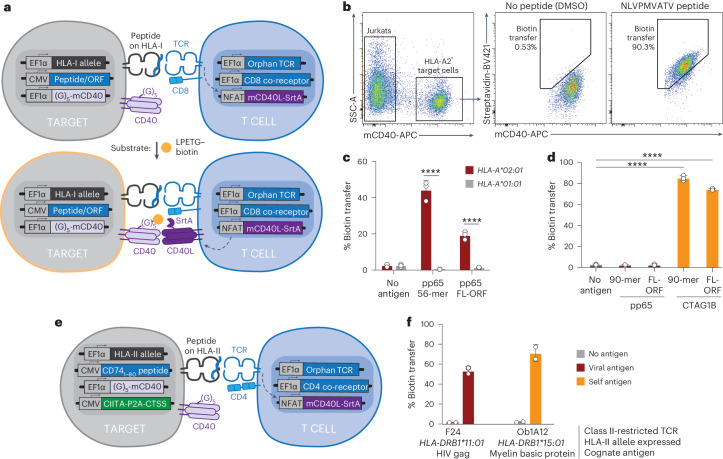
TCR-MAP efficiently and selectively identifies cognate human TCR–pMHC interactions. **a**, Schematic of the TCR-MAP antigen discovery method. Target APCs expressing HLA alleles of interest, the TCR-MAP biotin acceptor (G_5_-mCD40) and peptides or proteins of interest were cocultured with Jurkat cells transduced with TCRs of interest, coreceptors and the NFAT-inducible SrtA reporter (mCD40L–SrtA). Upon TCR activation, mCD40L–SrtA was expressed on the cell surface of Jurkats and covalently transferred LPETG–biotin substrates to cognate target cell acceptors. **b**, Representative flow cytometry plots of HLA-A2^+^ G_5_-target cells pulsed with or without the NLVPMVATV peptide cocultured with NLV3 TCR^+^ SrtA-Jurkat cells. Antigen recognition was assessed using streptavidin fluorophores to label biotinylated target cells. **c**, Quantification of overall target cell biotinylation when G_5_-targets expressed either the restricted (HLA-A*02:01) or nonrestricted (HLA-A*01:01) HLA allele and were transduced with either a 56-aa polypeptide containing the pp65 antigen NLVPMVATV or the full-length protein (FL-ORF) and cocultured with NLV3 TCR^+^ SrtA-Jurkat cells. ****P < 0.0001 for each group relative to the nonrestricted HLA allele control, determined by one-way ANOVA with a Tukey–Kramer multiple-comparison test. **d**, Quantification of target cell biotinylation when cocultured with the indicated antigen-positive target cell and TCR^+^ SrtA-Jurkat. ****P < 0.0001 for each group relative to the no-antigen control, determined by a two-tailed *t*-test. **e**, Schematic of TCR-MAP for class II-restricted TCRs. **f**, Quantification of target cell biotinylation when cocultured with the indicated antigen-positive target cell and TCR^+^ SrtA-Jurkat for class II-restricted T cells. Each dot in **c**, **d** and **f** represents a different biological replicate, where error bars indicate the mean and s.d. The data in **b**–**d** and **f** are representative of *n* = 3 independent biological replicates.

**Extended Data Fig. 1:**
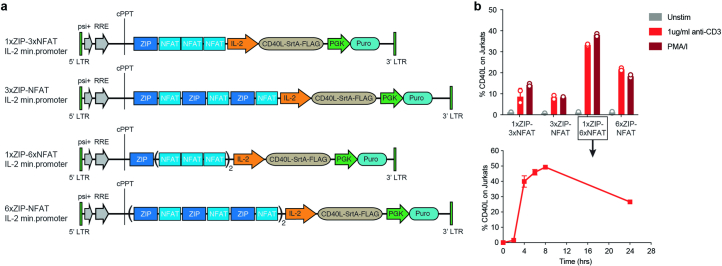
Design of an inducible SrtA expression construct for TCR-MAP. (**a**) Vector designs for the different inducible NFAT-SrtA reporters tested. (**b**) Jurkat cells transduced with the various NFAT-SrtA reporters were stimulated for 6 h (top) or 1, 4, 6, 8, 24 h (bottom) with either 1 μg/ml anti-CD3 antibody (top & bottom) or PMA/I (top). Percentage of murine CD40L expressed on the cell surface of Jurkats was assessed by flow cytometry. Error bars indicate the mean and SD. Data in b are representative of n = 3 independent biological replicates. PMA, phorbol 12-myristate 13-acetate. I, ionomycin.

To establish a highly sensitive and specific reporter system that would capture cognate TCR–pMHC interactions, we selected a previously reported proximity labeling strategy using the *Staphylococcus aureus* transpeptidase SrtA, which covalently transfers substrates containing the polypeptide motif LPXTG to nearby N-terminal oligoglycine residues^16,17^. We designed a two-cell method consisting of immortalized Jurkat T cells expressing a genetically fused mouse CD40 ligand–SrtA (mCD40L–SrtA) construct under an inducible nuclear factor of activated T cells (NFAT) promoter to serve as the donor (SrtA-Jurkats) (Extended Data Fig. 1a) and human leukocyte antigen (HLA) class I-null HEK-293T APCs transduced with an N-terminal oligoglycine-tagged mouse CD40 receptor (G_5_-mCD40) as the SrtA substrate acceptor (G_5_-targets)^17,18^ (Fig. 1a). Jurkat cells can be easily engineered to express TCRs of interest and additional TCR signaling components such as the CD8 coreceptor. Target cells can be further transduced with the desired MHC alleles and antigens encoded as peptide fragments or full-length proteins (FL-ORF (open reading frame)) for presentation. Upon T cell activation, mCD40L–SrtA is induced on the Jurkat cell surface and catalyzes the transfer of exogenously added LPETG–biotin substrates onto cognate target cells expressing the G_5_-mCD40 acceptor (Fig. 1a and Extended Data Fig. 1b). This method, which we call TCR-MAP, relies on a TCR-stimulated circuit in immortalized T cells to activate sortase-mediated biotinylation of cognate APCs.

To examine the specificity of TCR-MAP, we introduced the NLV3 TCR, specific for the human cytomegalovirus (CMV)-derived pp65 epitope, NLVPMVATV, into TCRβ-null SrtA-Jurkats and cultured them with HLA-A2^+^ G_5_-target cells that were pulsed with either DMSO or the pp65 epitope^13,19^. As assessed by flow cytometry, biotinylation of HLA-A2^+^ G_5_-targets occurred only in the presence of the cognate pp65 peptide (Fig. 1b). We next sought to determine whether genetically encoded peptide fragments or FL-ORFs could be processed and presented by the target cells for recognition by NLV3 TCR^+^ Jurkats. We expressed either a 56-aa (amino acid) fragment that contained the NLV epitope or the pp65 FL-ORF (561 aa) in HLA-A2^+^ G_5_-targets and found that both constructs resulted in biotinylation of HLA-A2^+^ target cells (Fig. 1c). The biotin signal was specific to target cells expressing the correct HLA class I-restricting allele and not HLA-A1^+^ G_5_-targets that expressed the nonrestricting allele (Fig. 1c). To assess the utility of the system beyond viral antigen–TCR pairs, we generated HLA-A2^+^ G_5_-target cells that encoded a 90-aa fragment from the cancer/testis antigen 1B (CTAG1B/NY-ESO-1) protein containing the IG4 TCR-specific epitope, SLLMWITQC^20^. SrtA-Jurkats transduced with the IG4 TCR specifically biotinylated HLA-A2^+^ G_5_-target cells only when the CTAG1B antigen (either the 90-aa peptide or the 180-aa FL-ORF) was expressed and showed no reactivity to the controls with no antigen or irrelevant antigens (Fig. 1d).

After establishing TCR-MAP for class I-restricted TCR–pMHC pairs, we next sought to engineer an equivalent method for class II-restricted TCRs based on the TScan-II peptide delivery strategy^15^. First, to ensure efficient processing and presentation of class II antigens, we transduced target cells with CIITA, the MHC class II transactivator, and CTSS, the cathepsin serine protease required for class II invariant chain processing^15,21–24^. Next, we used transient CRISPR (clustered regularly interspaced short palindromic repeats) with Cas9 nucleofection to mutate all class II alleles using single guide RNAs (sgRNAs) targeting the HLA-DR, HLA-DP and HLA-DQ class II locus. Lastly, to direct antigens to the MHC class II-containing cellular compartments, oligonucleotides encoding peptide antigens were genetically fused downstream of a truncated N-terminal sequence of CD74 (invariant chain) for lentiviral expression in target cells^5,8,15,25^ (Fig. 1e). SrtA-Jurkat cells were modified to express the CD4 coreceptor and either F24 or Ob1A12 TCRs, which recognize peptide antigens derived from the human immunodeficiency virus (HIV) Gag polyprotein (DR11^+^/Gag293_299__–__312_/RFYKTLRAEQASQE) or myelin basic protein (DR15^+^/MBP_85__–__99_/ENPVVHFFKNIVTPR), respectively^26–28^. In alignment with the results obtained for HLA-A2-restricted TCR responses, G_5_-target cells expressing the correct HLA class II allele and antigen were specifically recognized and biotinylated (Fig. 1f). These data demonstrate that TCR-MAP is a specific and sensitive system to capture cognate pMHC–TCR interactions for both class I-restricted (HLA-A2) and class II-restricted T cells in humans.

### TCR-MAP captures mouse MHC class I-restricted (H2-K^b^) and class II-restricted (H2-IA^b^) pMHC–TCR interactions

**Fig. 2:**
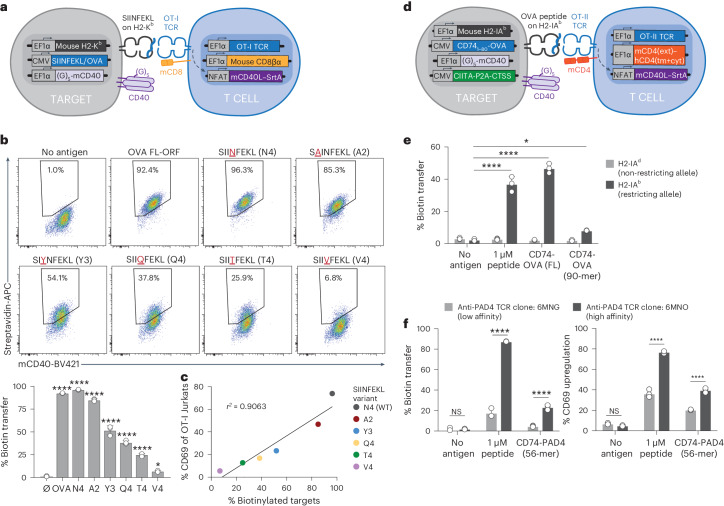
TCR-MAP quantitatively captures class I-restricted and class II-restricted cognate mouse TCR–pMHC interactions according to TCR affinity. **a**, Schematic of TCR-MAP to enable mouse antigen presentation and TCR signaling. **b**, Full-length OVA and SIINFEKL peptide variants expressed in H2-K^b^^+^ G_5_-target cells and cocultured with OT-I TCR^+^ SrtA-Jurkats. Representative flow plots and quantification of target cell biotinylation. *P = 0.0292 and ****P < 0.0001 for each group relative to the no-antigen control, determined by a two-tailed *t*-test. **c**, The relative correlation between CD69 upregulation of OT-I TCRs (*y* axis) and biotinylated target cells (*x* axis) upon coculture. The *r*^*2*^ value is reported. **d**, Schematic of mouse class II-restricted TCR antigen discovery. **e**, Quantification of overall target cell biotinylation when CIITA^+^CTSS^+^ G_5_-targets expressed either the restricted (H2-IA^b^) or the nonrestricted (H2-IA^d^) mouse MHC allele and were pulsed with the OVA class II epitope, KISQAVHAAHAEINEAG, or transduced with either full-length OVA or the 56-aa polypeptide containing the class II OVA epitope fused to the N terminus of the invariant chain (CD74). *P = 0.0124 and ****P < 0.0001 for each group relative to the no-antigen control, determined by one-way ANOVA with a Tukey–Kramer multiple-comparison test. **f**, Quantification of target cell biotinylation (left) and CD69 upregulation (right) for cocultures testing for differential reactivity between high-affinity (6MNO) and low-affinity (6MNG) TCRs reactive against the PAD4 antigen. ****P < 0.0001 for each group relative to the low-affinity TCR condition, determined by one-way ANOVA with a Tukey–Kramer multiple-comparison test. Each dot in **b**, **e** and **f** represents a different biological replicate, where error bars indicate the mean and s.d. The data in **b**, **c**, **e** and **f** are representative of *n* = 3 independent biological replicates. NS, not significant.

Given the success of TCR-MAP in distinguishing cognate antigen–TCR specificities from humans, we next sought to test the method’s ability to assess mouse TCRs using model TCR–antigen pairs. We chose the well-characterized OT-I TCR–SIINFEKL epitope pair and established G_5_-target cells that coexpressed the mouse H2-K^b^ MHC class I allele with full-length ovalbumin (OVA) or the minimal SIINFEKL epitope. To our TCRβ-null SrtA-Jurkats, we cotransduced the mouse CD8 coreceptor and the OT-I TCR, which recognizes the 8-aa SIINFEKL epitope derived from OVA (Fig. 2a). Coculture of the two engineered cells showed robust biotinylation of G_5_-target cells, verifying that ectopic expression of mouse MHC alleles, coreceptors and TCRs into immortalized human cell lines was sufficient for antigen recognition (Fig. 2b). To study how TCR affinity for an antigen impacts the overall signal-to-noise ratio of TCR-MAP, we next tested the reactivity of the OT-I TCR^+^ SrtA-Jurkats against mutant variants of the known SIINFEKL (N4) epitope^29^. We selected five variants with equivalent binding affinity to H2-K^b^ but which differed in their overall ability to stimulate OT-I T cells^29^. We observed a direct correlation between target cell biotinylation and TCR reactivity for the SIINFEKL variant series tested (Fig. 2c).

With the success of the OT-I/SIINFEKL system, we next asked whether a parallel approach could be taken to establish TCR-MAP for class II-restricted mouse CD4^+^ TCR–antigen pairs. To achieve this goal, we first transduced the HLA class II-null, CIITA^+^CTSS^+^ G_5_-target cells with the mouse H2-IA^b^ class II allele and full-length OVA fused downstream of the truncated invariant chain construct (CD74–OVA). Next, we transduced TCRβ-null SrtA-Jurkats with the OT-II TCR and a chimeric CD4 coreceptor composed of the extracellular mouse CD4 domain fused to the transmembrane and cytoplasmic region of human CD4 (Fig. 2d). Coculture of the two engineered target and Jurkat cell lines demonstrated significant biotin transfer that was specific for the restricting H2-IA^b^ allele-expressing target cells but not the nonrestricting H2-IA^d^ allele expressors or target cells that did not express the antigen (Fig. 2e). As expected, peptide pulsing and expression of a 90-aa tile that contained the known OT-II epitope (KISQAVHAAHAEINEAG; CD74–OVA (90-mer)) also resulted in target cell biotinylation that was restricted to H2-IA^b^ (Fig. 2e), although the degree to which APCs were biotinylated varied depending on the length of the genetically encoded OVA antigen. Lastly, to assess the sensitivity and ability of the engineered mouse class II system for self-antigen discovery, we generated SrtA-Jurkats that expressed two distinct mouse TCRs that were each reactive against the same antigen, mouse peptidyl arginine deiminase 4 (PAD4), but which differed in their affinity for the cognate target^30^. Similar to the SIINFEKL variant experiment, TCR-MAP nicely phenocopied the known TCR affinities for PAD4; the SrtA-Jurkats transduced with the higher-affinity anti-PAD4 TCR (clone 6MNO) exhibited significantly stronger G_5_-target cell biotinylation and Jurkat cell activation through CD69 upregulation when compared to the known lower-affinity anti-PAD4 TCR (clone 6MNG) (Fig. 2f). Overall, our data demonstrate that TCR-MAP is readily adaptable to study mouse class I-restricted (H2-K^b^) and class II-restricted (H2-IA^b^) TCR interactions and the magnitude of the signal is determined by the strength of the TCR–ligand interaction.

### Virome-wide screens and TCR binding footprints with TCR-MAP

Mapping the exact specificity of a given TCR in terms of both the antigen recognized and its nuanced binding footprint is important for our understanding of how and what pathogens or autoantigens are recognized by T cells. Having shown that TCR-MAP cleanly discriminates cognate versus noncognate interactions and sensitively captures a wide range of TCR affinities against genetically encoded peptides, we reasoned that the method would be able to identify T cell antigens from complex, oligonucleotide libraries and map epitope binding footprints of TCRs.

**Extended Data Fig. 2:**
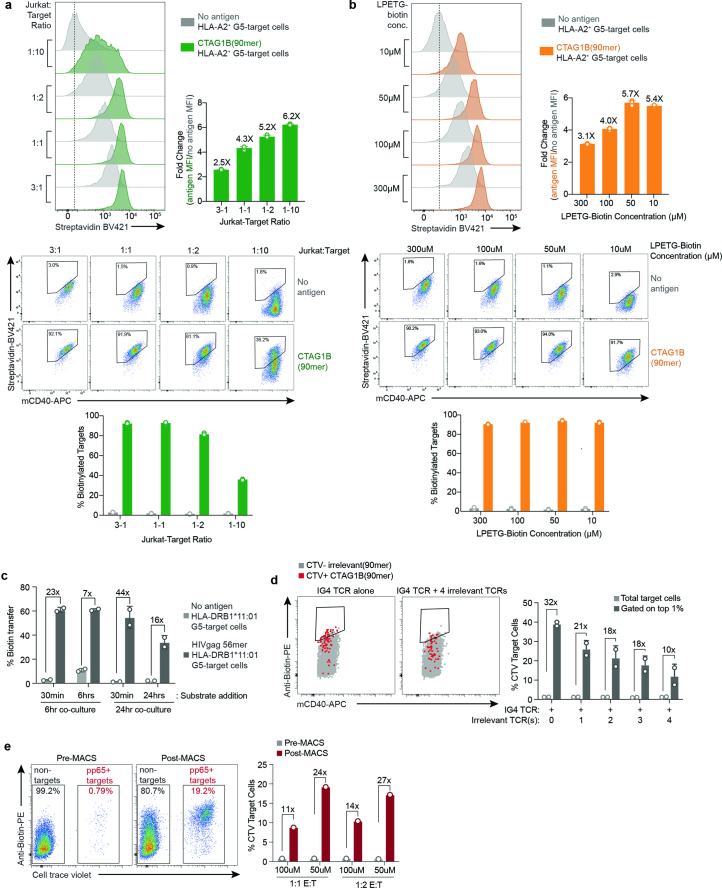
Optimization of the TCR-MAP platform. (**a**) Streptavidin cell surface labeling of HLA-A*02:01^+^ G_5_-target cells transduced with (green) or without (gray) the CTAG1B antigen at the indicated effector-to-target cell ratios with IG4 TCR^+^ SrtA-Jurkats for 6 h using an LPETG-biotin concentration of 50 μM. Reported fold change values were calculated based on the ratio of streptavidin fluorophore MFI of the CTAG1B 90-aa peptide expressing G_5_-targets over streptavidin fluorophore MFI of no antigen controls. (**b**) Streptavidin cell surface labeling of HLA-A*02:01^+^ G_5_-target cells transduced with (orange) or without (gray) the CTAG1B antigen at the indicated concentration of LPETG-biotin substrate added to a co-culture with IG4 TCR^+^ SrtA-Jurkats for 6 h at an effector-to-target ratio of 1:1. Reported fold change values (Right) were calculated based on the ratio of streptavidin fluorophore MFI of the CTAG1B 90-aa tile expressing G_5_-targets over streptavidin fluorophore MFI of no antigen controls on the bottom. (**c**) Quantification of HLA-DRB1*11:01^+^ CIITA^+^CTSS^+^ G_5_-target cell biotinylation after transduction with HIV gag 56-aa fragment containing the known antigen for F24 TCR. LPETG-biotin substrate (50 μM) was added at the indicated times. Reported fold change values were calculated based on the ratio of the mean of antigen-expressing G_5_-target cell biotinylation over the mean of no antigen G_5_-target cell biotinylation. Co-cultures were performed at an effector-to-target ratio of 1:1 (**d**) Representative flow plots looking at target cell biotinylation of HLA-A*02:01^+^ G_5_-target cells transduced with (red) or without (gray) the CTAG1B antigen. CTV labeled antigen^+^ G_5_-target cells were spiked into non-antigen controls at a frequency of 1% and co-cultured with IG4 TCR^+^ SrtA-Jurkats alone or combined with four other non-cognate TCRs at a final effector-to-target ratio of 1:1 for 6 h using an LPETG-biotin concentration of 50 μM. Quantification of the total frequency of CTV labeled antigen^+^ G_5_-targets relative to the whole population (light gray) or gated on the top 1% of biotinylated G_5_-targets (dark gray) is reported for all TCR pooling conditions. Reported fold change values were calculated based on the ratio of the frequency of CTV^+^ G_5_-target cells in the top 1% of biotinylated cells over their frequency in the total input population. (**e**) Representative flow plots showing the pre- and post-magnetic bead enrichment of target cells. Pp65 antigen^+^ HLA-A*02:01^+^ G_5_-target cells were spiked into no antigen control cells at a frequency of <1% and co-cultured with NLV3 TCR^+^ SrtA-Jurkats for 6 h using an LPETG-biotin concentration of 50 μM at an effector-to-target ratio of 1:1 or 1:2, as indicated. Cells from the co-culture were labeled with streptavidin conjugated magnetic beads and run over a magnetic enrichment column (Miltenyi). Cell bound to the column were eluted and analyzed by flow cytometry to quantify cognate antigen (CTV^+^) enrichment. Reported fold change values were calculated based on the ratio of the frequency of CTV^+^ G_5_-target cells bound to the column versus their frequency in the input population. Each dot in c-e represents a different biological replicate, where error bars in a-e indicate SD. Data in a-e are representative of n = 3 independent biological replicates. MFI, mean fluorescence intensity. CTV, cell trace violet.

We first empirically tested several conditions to determine the optimal signal-to-noise ratio of TCR-MAP, including effector-to-target cell ratios, concentration of the LPETG–biotin substrate and coculture timing (Extended Data Fig. 2a–c). We found that the biotin signal was significantly maintained on G_5_-target cells even up to 24 h after coculture and maintaining low Jurkat cell-to-target ratios improved the signal-to-noise ratio of TCR-MAP. Next, to mimic a pooled genetic screening situation where cognate target cells would be a small fraction of a large library, we performed spike-in experiments where we labeled cognate G_5_-target cells with cell trace violet and added them at a frequency of <1% to controls with either no antigen or irrelevant antigens and queried their relative enrichment with a single TCR or multiple pooled TCRs (Extended Data Fig. 2d). Even when TCRs were multiplexed, cognate G_5_-targets were enriched within the top 1% of all biotinylated targets. Lastly, we determined whether antigens were able to be enriched using magnetic beads and found that cognate G_5_-target cells were enriched 10–30-fold relative to the input frequencies (Extended Data Fig. 2e).

**Fig. 3:**
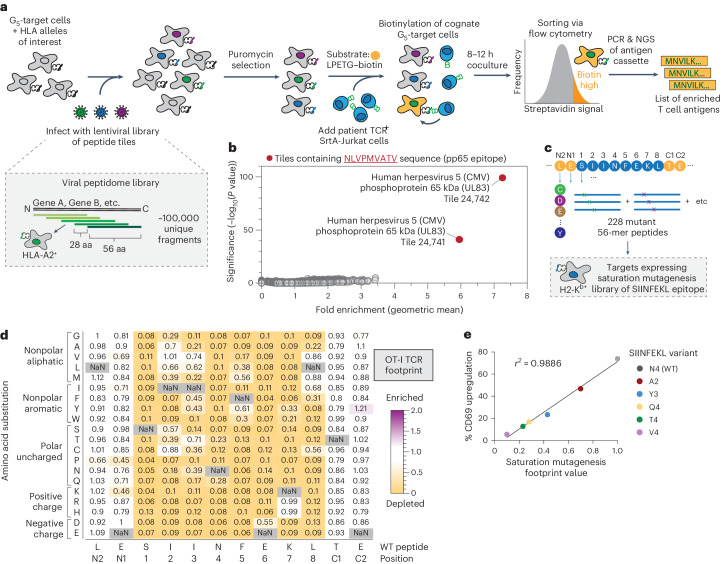
TCR-MAP accurately identifies cognate T cell antigens from complex, pooled library screens and generates high-resolution epitope binding footprints of TCRs. **a**, Workflow of TCR-MAP antigen discovery pipeline. Engineered target cells were infected with a lentivirus encoded peptide library, selected with antibiotics and cultured with SrtA-Jurkats expressing TCRs of interest in the presence of LPETG–biotin substrate. After 8–12 h, biotinylated target cells were isolated by fluorescence-activated cell sorting (FACS), and peptide-encoding DNA sequences were PCR-amplified from gDNA. Sequencing and analysis of enriched reads relative to the input library enabled the calculation of adjusted *P* values to identify enriched peptides. **b**, NLV3 TCR^+^ SrtA-Jurkats were screened against a genome-wide viral library in HLA-A*02:01^+^ G_5_-target cells. Each dot represents one peptide, with the *y* axis indicating the −log_10_ adjusted *P* values determined by Mageck and the *x* axis indicating the geometric mean of the enrichment of the peptide across three replicates. Fold enrichment is defined as the ratio of the abundance of the peptide in the sorted population relative to the input library. Peptides highlighted in red contain the known cognate antigen for the NLV3 TCR. **c**, Design of the SIINFEKL epitope saturation mutagenesis library. Each position was substituted to each of the 19 alternative amino acids. In addition to the epitope, the two amino acids that were N terminal and C terminal to the epitope were examined. Each mutant epitope was expressed as 56-aa peptide tiles, introduced into H2-K^b^^+^ G_5_-target cells and screened using OT-I TCR^+^ SrtA-Jurkats using TCR-MAP. **d**, Heatmap representing the relative recognition of mutant epitopes in the saturation mutagenesis library relative to the unsubstituted SIINFEKL epitope by the OT-I TCR. Each box in the heatmap shows the relative enrichment or depletion of a single mutant peptide, where the amino acid along the *x* axis was substituted to the amino acid indicated along the *y* axis. NaN, not a number. **e**, The relative correlation of the saturation mutagenesis footprint values is plotted versus the CD69 upregulation of OT-I TCRs by that same epitope sequence and the *r*^*2*^ value is reported. Source data

To test the sensitivity of TCR-MAP against a large library, we performed proof-of-concept screens using the well-characterized NLV3 TCR against the viral peptidome library consisting of ~100,000 unique oligonucleotides encoding 56-aa fragments offset by 28 aa across all proteins derived from viral species known to infect humans^13^. This library contains two 56-aa tiles encoding the pp65 epitope to serve as positive controls. After library introduction and puromycin selection, viral peptidome-expressing HLA-A2^+^ target cells were cultured with NLV3 TCR^+^ SrtA-Jurkat cells in the presence of LPETG–biotin. We sorted the top 1% of biotinylated target cells on the basis of streptavidin fluorophore labeling and identified the enriched antigens by Illumina sequencing by comparing the relative abundance of each oligonucleotide before and after the sort (Fig. 3a). Notably, even in the context of a large, diverse library of ~100,000 unique fragments, the top two enriched oligonucleotide tiles were the adjacent 56-aa fragments that encoded the CMV-derived pp65 epitope, NLVPMVATV (Fig. 3b).

**Extended Data Fig. 3:**
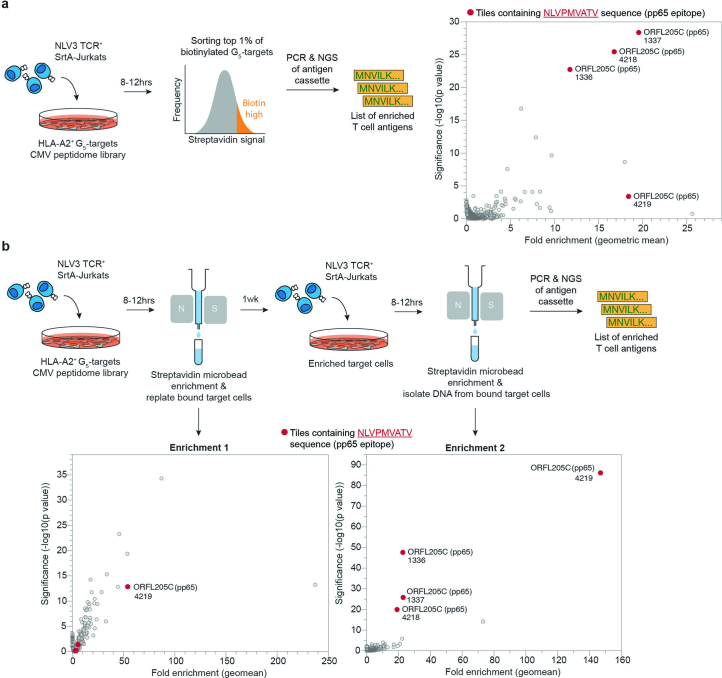
Magnetic bead enrichment is capable of isolating cognate antigens using TCR-MAP. TCR-MAP screen schematic and results of NLV3 TCR^+^ SrtA-Jurkats screened against a CMV-specific viral peptidome library in HLA-A*02:01^+^ G_5_-target cells. Biotinylated G_5_-target cells were either FACS isolated (**a**) or labeled with streptavidin conjugated magnetic beads and run over a magnetic enrichment column for a total of two consecutive purifications for isolation (**b**). Cellular barcodes from isolated cells are PCR amplified from gDNA. Downstream NGS and analysis of enriched reads relative to the input library enables calculation of adjusted p values to call enriched peptides by the TCR queried. The results of the screen are plotted such that each dot represents one peptide with the y axis indicating the negative log10 p adjusted values by Mageck and the x axis reporting the geometric mean of the enrichment of the peptide across three replicates. Fold enrichment is defined as the ratio of the abundance of the peptide in the sorted population relative to the input library. Peptides highlighted in red contain the known cognate antigen for the NLV3 TCR. CMV, cytomegalovirus. FACS, fluorescence-activated cell sorting. gDNA, genomic DNA. Source data

Having shown that sorting the top 1% of biotinylated targets results in sensitive identification of cognate antigens, we next tested whether rounds of streptavidin magnetic bead purification could also enrich for cognate antigens. We screened the NLV3 TCR against a 56-aa fragment library tiling the entire CMV proteome (5,764 unique peptides) and found that two rounds of enrichment were sufficient to capture the four fragments in the library that contained the antigenic epitope with a signal-to-noise ratio comparable to sorting (Extended Data Fig. 3a,b). Antigen discovery screens using TCR-MAP can, thus, be performed without reliance on flow cytometry sorters.

Lastly, to test whether we could build high-resolution TCR–peptide binding footprints using TCR-MAP, we screened a comprehensive saturation mutagenesis library for the OT-I TCR–SIINFEKL antigen pair (Fig. 3c). This single mutant library was composed of 56-aa tiles where the SIINFEKL epitope and two amino acids immediately upstream and downstream of the antigen were substituted to each of the 19 alternative amino acids (Fig. 3c). We compared the enrichment of each mutant in the library to the wild-type (WT) SIINFEKL peptide tiles and generated a critical binding interface heatmap based on the relative enrichment or depletion scores (Fig. 3d). As expected, the vast majority of substitutions to the SIINFEKL epitope resulted in abrogated or decreased OT-I TCR recognition (Fig. 3d). However, substitutions to amino acids with similar chemical properties were tolerated at several positions, such as positions 7 and 8 (Fig. 3d). Because we previously showed that TCR-MAP can capture a wide range of OT-I TCR affinities to mutant variants of SIINFEKL (Fig. 2b), we correlated the saturation mutagenesis footprint values obtained from the screen to the relative ability of individual SIINFEKL variants to stimulate OT-I TCRs. The results of the SIINFEKL saturation mutagenesis screen showed strong concordance with the known antigenic stimulatory capacity of the SIINFEKL variants (Fig. 3e). Thus, TCR-MAP is a powerful method that can isolate and decode cognate antigen-expressing target cells from a complex, pooled library setting and capture TCR–epitope binding footprints using saturation mutagenesis antigen libraries with high sensitivity and accuracy.

### Genome-wide TCR-MAP screens using single or pooled TCRs

Beyond TCR specificities to foreign sources such as viruses, self-antigens comprise another class of important antigenic targets. Particularly in cancer, boosting T cell responses against cancer-expressed testis antigens has shown promising antitumor responses clinically and efforts aimed at identifying TCRs with high on-tumor and low off-tumor specificity is an active area of research. However, the task of mapping self-reactivities is difficult because of the vast size of the human proteome from which antigens can arise and because of the often-low TCR affinities to self-peptides^3^.

**Fig. 4:**
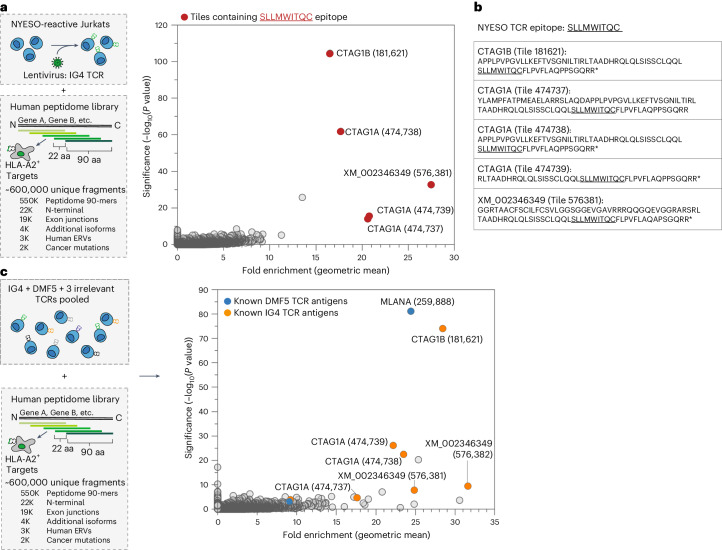
Genome-wide TCR-MAP screens can be performed using single or pooled TCRs. **a**, Schematic of the human genome-wide TCR-MAP screen of the IG4 TCR^+^ SrtA-Jurkats and peptide enrichment results. The 90-aa peptides tiled across the human proteome with 22-aa overlap were expressed in HLA-A*02:01^+^ G_5_-target cells. Each dot represents one peptide, with the *y* axis plotting the −log_10_ adjusted *P* values determined by Mageck and the *x* axis indicating the geometric mean of the enrichment of the peptide across three replicates. Fold enrichment is defined as the ratio of the abundance of the peptide in the sorted population relative to the input library. Peptides highlighted in red contain the known epitope sequence for the IG4 TCR. **b**, List of the peptide fragments that scored in the IG4 TCR screen, where the known epitope is underlined within the peptide fragment. **c**, Schematic of the human genome-wide TCR-MAP screen pooling five TCR^+^ SrtA-Jurkats at a time and peptide enrichment results. The 90-aa peptides tiling across the human proteome with 22-aa overlap were expressed in HLA-A*02:01^+^ G_5_-target cells. To the target cells, IG4, DMF5 and three HLA-A*02:01-restricted non-self-reactive TCR^+^ SrtA-Jurkats were added to achieve a final effector-to-target ratio of 1:1. Cells were cocultured for 8 h and biotinylated G_5_-target cells were isolated by FACS. The screening results are depicted as a dot plot, where each dot represents one peptide, with the *y* axis reporting the −log_10_ adjusted *P* values determined by Mageck and the *x* axis indicating the geometric mean of the enrichment of the peptide across three replicates. Fold enrichment is defined as the ratio of the abundance of the peptide in the sorted population relative to the input library. Peptides highlighted in blue contain the known epitope sequence for the DMF5 TCR, while the fragments highlighted in orange contain the known epitope sequence for the IG4 TCR. Source data

To test whether TCR-MAP would aid in T cell antigen discovery of self-reactive TCRs at a genome scale, we performed TCR-MAP screens using the class I-restricted CTAG TCR, IG4, against the human peptidome library^3,15,31^. In addition to full-length ORF proteins tiled with 90-aa peptides offset by 22 aa, this library includes additional N-terminal fragments to increase coverage of the beginning sequence of ORFs, additional protein isoforms and human endogenous retroviral proteins for a comprehensive library containing ~580,000 unique peptides (Fig. 4a). When IG4 TCR^+^ SrtA-Jurkats were screened against HLA-A2^+^ G_5_-target cells expressing the human peptidome library, the top enriched peptide tiles were the CTAG1B and CTAG1A fragments that contained the known antigenic 9-mer epitope (Fig. 4a,b). In addition, a single peptide tile from hypothetical protein XM_002346349 was significantly enriched with a similar magnitude (Fig. 4a). We found that the 90-aa fragment of XM_002346349 also contained the antigenic SLLMWITQC epitope (Fig. 4b), highlighting the utility and sensitivity of TCR-MAP to identify cognate and cross-reactive antigens from a large, complex peptidome library.

A major goal of T cell antigen discovery efforts is to map the reactivities of multiple TCRs at a time. To increase the throughput of our screens, we wondered whether SrtA-Jurkats expressing different TCR sequences could be pooled together without compromising the signal-to-noise ratio of the approach. We performed antigen discovery screens against the human peptidome combining SrtA-Jurkat cells expressing the cancer/testis antigen-reactive IG4 or DMF5 TCRs (A2^+^/melanocyte antigen (MLANA)/EAAGIGILTV), with three additional HLA-A2-restricted TCR clonotypes that do not exhibit reactivity to self-antigens contained in the human peptidome library (Fig. 4c). As expected, the CTAG1B, CTAG1A and XM_002346349 peptide fragments containing the IG4 TCR epitope, SLLMWITQC, showed comparable enrichment compared to the IG4 TCR when screened alone using TCR-MAP (Fig. 4a). A peptide fragment from MLANA containing the antigen for the DMF5 TCR, EAAGIGILTV, was also strongly enriched (Fig. 4c). Proteome-wide T cell antigen discovery screens via TCR-MAP can not only be used to search for TCR reactivities for one TCR at a time but can also be multiplexed to assess five or more TCRs, thus increasing the throughput of the system.

**Extended Data Fig. 4:**
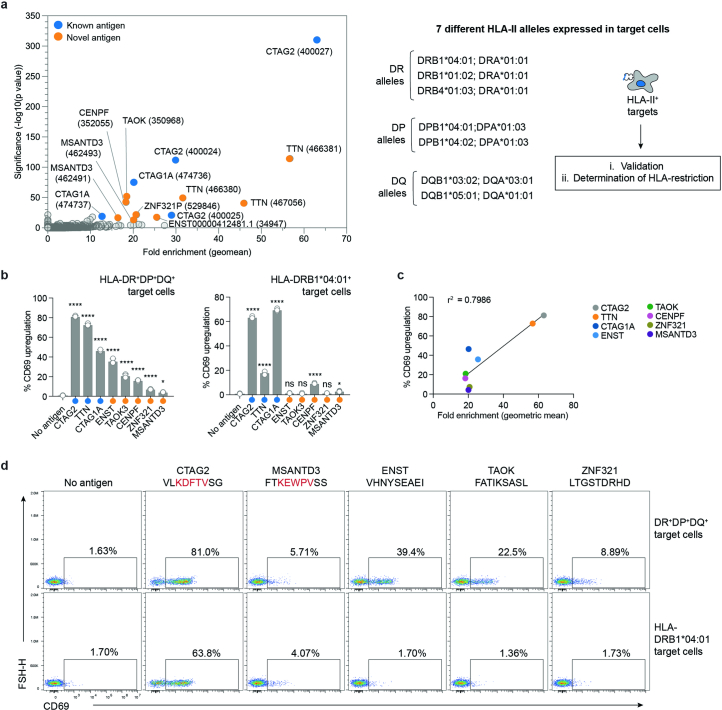
TCR-MAP identifies cross-reactive and cross-restrictive antigenic hits for CTAG-reactive CD4 TCR. (**a**) Human genome-wide TCR-MAP screen results of the TCR3898-2 TCR. Each dot represents one peptide with the y axis plotting the negative log10 p adjusted values by Mageck and the x axis calling the geometric mean of the enrichment of the peptide across three replicates. Fold enrichment is defined as the ratio of the abundance of the peptide in the sorted population relative to the input library. Peptides highlighted in blue are known antigens of the TCR3898-2 TCR, while yellow indicates new antigens that validated. (**b**) Determination of CD69 upregulation of TCR3898-2 TCRs to the indicated antigens in CIITA^+^CTSS^+^ G_5_-target cells that express six different HLA-DR, -DP, and -DQ alleles or only the HLA-DRB1*04:01 allele to assess HLA-restriction patterns. The relative correlation of the fold enrichment of the peptide fragment vs the CD69 upregulation of TCR3898-2 TCRs by that same epitope sequence is plotted in (**c**) and the r^2^ value reported. (**d**) Representative flow cytometry plots of 3898-2 TCR^+^ SrtA-Jurkat cell activation by assessing CD69 upregulation in response to the indicated peptides expressed in CIITA^+^CTSS^+^ G_5_-target cells expressing all six DR^+^DP^+^DQ^+^ alleles or only the HLA-DRB1*04:01 allele. * p = 0.0266 (left) and 0.0397 (right), **** p < 0.0001 for each group relative to no antigen control by two-tailed t test. Each dot in b represents a different biological replicate, where error bars indicate the mean and SD. Data in e are representative of n = 3 independent biological replicates. HLA, human leukocyte antigen. CIITA, class II transactivator. CTSS, cathepsin S. Source data

Thus far, we demonstrated the ability of TCR-MAP to determine TCR reactivities in the context of single HLA allele-expressing target cells. However, the cells of our body can express up to eight different HLA class I or class II alleles. Moreover, promiscuous class II-restricted CD4^+^ T cells have been known to exhibit both cross-reactive (recognition of different antigens on a common HLA class II allele) and/or cross-restrictive behavior (single TCR recognizing two different antigens on two different HLA class II alleles)^32–34^. We next screened TCR3898-2, which recognizes an epitope from CTAG1B (DR4^+^/CTAG1B_121__–__130_/VLLKEFTVSG), against CIITA^+^CTSS^+^ G_5_-target cells expressing six different HLA class II alleles, including the restricting HLA-DRB1*04:01 allele, transduced with the invariant chain fused human peptidome library (Extended Data Fig. 4a). As expected, several 90-aa fragments from the human peptidome library containing the known antigenic epitope from CTAG1A, the close family member CTAG2 and TTN scored (Extended Data Fig. 4a)^15^. Notably, several additional 90-aa fragments from new protein sources including TAOK (a serine/threonine protein kinase), CENPF (centromere protein F), ZNF321 (zinc finger protein 321) and MSANTD3 *(*Myb/SANT-like DNA-binding domain-containing protein 3) and a single N-terminal peptide (ENST00000412481.1) were also enriched in the screen (Extended Data Fig. 4a). To validate these hits, we generated G_5_-target cells that expressed each tile individually and tested for Jurkat cell activation by CD69 upregulation. Although TCR3898-2 was previously characterized to not exhibit cross-reactivity or cross-restriction^31^, the peptide fragments did indeed validate (Extended Data Fig. 4b,d). Moreover, individual fragments showed excellent concordance with fold enrichment values from the human peptidome screen, demonstrating that TCR-MAP sensitively captures antigen hierarchies of varying TCR strength (Extended Data Fig. 4c). To assess which of the validated fragments were restricted specifically to the HLA-DRB1*04:01 allele relative to the other expressed HLA class II alleles, we generated target cells that coexpressed HLA-DRB1*04:01 and each of the individual 90-aa fragments (Extended Data Fig. 4b,d). From the Jurkat cell activation data, we concluded that peptide tiles from CTAG2, TTN, CTAG1A, CENPF and MSANTD3 were restricted to HLA-DRB1*04:01, while antigenic peptides from ENST, TAOK and ZNF321 were likely presented by one of the other HLA class II alleles. In alignment with these results, the known LKEF sequence motif recognized by the TCR3898-2 CD4^+^ TCR or a very similar epitope sequence was contained only in the validated DR4-restricted peptide tiles (Extended Data Fig. 4d)^15,31^. TCR-MAP is a powerful tool to detect cognate antigen reactivities that may be cross-reactive and/or cross-restrictive.

### TCR-MAP discovers autoantigens of mouse TCRs

**Fig. 5:**
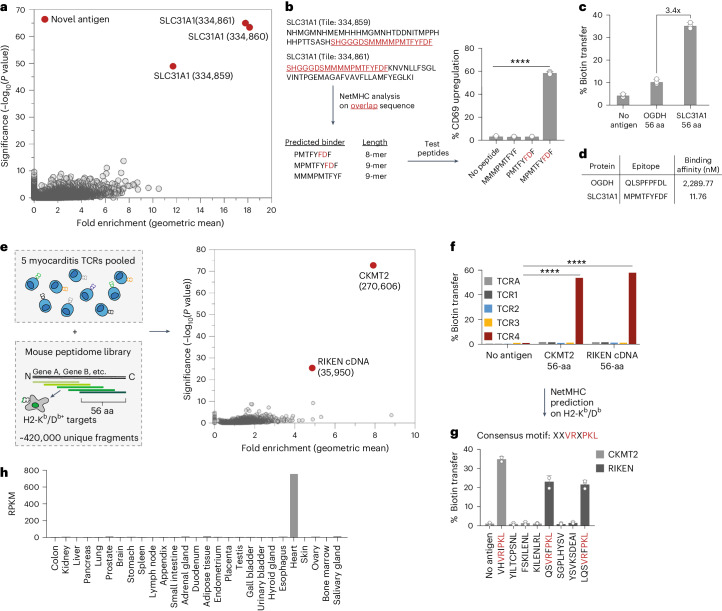
Genome-wide screens using TCR-MAP discovers autoantigen reactivities of mouse TCRs. **a**, Mouse genome-wide TCR-MAP screen results of the 2C TCR. The 56-aa peptide fragments covering the mouse proteome were expressed in H2-L^d^^+^ G_5_-target cells. Each dot in **a** and **e** represents one peptide, with the *y* axis plotting the −log_10_ adjusted *P* values determined by Mageck and the *x* axis calling the geometric mean of the enrichment of the peptide across three replicates. Fold enrichment is defined as the ratio of the abundance of the peptide in the sorted population relative to the input library. **b**, List of the overlapping fragments that scored in the 2C TCR screen, where the common peptide sequence between the two tiles is underlined. NetMHC was subsequently run to predict three possible 8-aa or 9-aa peptide binders on H2-L^d^; these peptides were pulsed on H2-L^d^^+^ G_5_-target cells and 2C TCR^+^ SrtA-Jurkat reactivity was assessed. ****P < 0.0001 for each group relative to the no-antigen control, determined by a two-tailed *t*-test. **c**, Quantification of H2-L^d^^+^ G_5_-target cell biotinylation expressing 56-aa peptide tiles containing the known antigen for OGDH or SLC31A1. **d**, Table of NetMHC binding affinity predictions for the indicated epitopes from OGDH or SLC31A1 on the H2-L^d^ allele. **e**, Mouse genome-wide TCR-MAP screen results of five mouse expanded myocarditis-specific TCRs. H2-K^b^/D^b^^+^ G_5_-target cells were cultured with multiplexed SrtA-Jurkat cells at an effector-to-target ratio of 1:1. **f**, Individual TCRs were tested against 56-mer tiles from CKMT2 and RIKEN cDNA. ****P < 0.0001 for each group relative to the no-antigen control for the specific TCR, determined by one-way ANOVA with a Tukey–Kramer multiple-comparison test. **g**, Predicted H2-K^b^ or H2-D^b^ peptide binders were pulsed into G_5_-target cells, cocultured with TCR4^+^ SrtA-Jurkats and assessed for biotinylation. **h**, CKMT2 RPKM (reads per kilobase of transcript per million reads mapped) values from an RNA-seq dataset on 27 human tissues or organs^43^. Each dot in **b**, **c** and **g** represents a different biological replicate, where error bars indicate the mean and s.d. Data in **b**, **c**, **e** and **g** are representative of *n* = 3 independent biological replicates. Source data

Although genome-scale antigen discovery methods have been described for the study of human TCRs, fewer high-throughput approaches exist to query TCRs from mice^6,35^. We next wondered whether TCR-MAP could be applied to discover T cell antigens for class I-restricted (H2-K^b^) and class II-restricted (H2-IA^b^) mouse TCRs. To test this, we selected the well-characterized 2C TCR that has a known H2-L^d^-associated peptide antigen, QLSPFPFDL (QL9), derived from the enzyme ɑ-ketoglutarate dehydrogenase (OGDH)^36,37^. G_5_-target cells expressing mouse H2-L^d^ were transduced with a mouse peptidome library encoding 56-aa fragments and screened against 2C TCR^+^ SrtA-Jurkat reporter cell lines. To our surprise, three overlapping peptide fragments derived from a high-affinity copper transporter membrane protein, SLC31A1, were the only tiles that significantly enriched (Fig. 5a). To narrow down the antigenic epitope within the validated hits, we performed NetMHC^38^ analysis on the overlapping polypeptide sequence from the adjacent peptide tiles from SLC31A1 and assessed the presence of H2-L^d^ binders (Fig. 5b). We tested the top predicted H2-L^d^ 8-aa and 9-aa binders by performing peptide pulse experiments and determined the 9-mer peptide, MPMTFYFDF, to be the reactive antigen for 2C TCR^+^ Jurkats (Fig. 5b). Notably, this epitope contained the FD motif, which is required for recognition by 2C TCR^39^. These results highlight the utility of comprehensive T cell antigen discovery screens using TCR-MAP as TCR cross-reactivities may be difficult to predict using homology-based searches with minimal motifs.

Although we validated SLC31A1 as an antigen for the 2C TCR, we wondered why the previously characterized antigen, OGDH, did not score in our screen. We generated H2-L^d^ target cells that expressed OGDH or SLC31A1 as 56-aa fragments and compared their relative reactivities (Fig. 5c). The peptide fragment from SLC31A1 activated 2C TCR^+^ SrtA-Jurkats with >3-fold greater potency than the peptide tile from OGDH (Fig. 5c). Further supporting the observed reactivities, epitope binding affinity predicted by NetMHC revealed that the epitope from SLC31A1 bound H2-L^d^ with ~200-fold greater affinity than that from OGDH (Fig. 5d). Thus, from our unbiased mouse peptidome screens, we identified a previously uncharacterized mouse self-antigen from SLC31A1 as a new and potent target of the 2C TCR.

**Extended Data Fig. 5:**
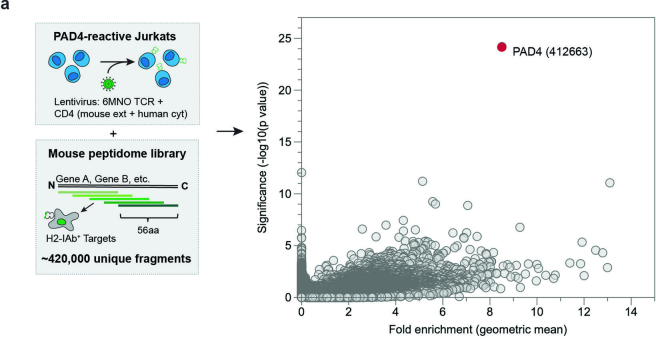
TCR-MAP accurately identifies Treg TCR reactivities. (**a**) Mouse genome-wide TCR-MAP screen results of PAD4-reactive 6MNO TCR^+^ SrtA-Jurkats. 56-aa peptide fragments covering the murine proteome were expressed in H2-IAb^+^ CIITA^+^CTSS^+^ G_5_-target cells SrtA-Jurkat cells were added to achieve a final effector-to-target ratio of 1:1. Each dot represents one peptide with the y axis reporting the negative log10 p adjusted values by Mageck and the x axis indicating the geometric mean of the enrichment of the peptide across three replicates. Fold enrichment is defined as the ratio of the abundance of the peptide in the sorted population relative to the input library. Peptides highlighted in red indicate validated hits from the screen. PAD4, peptidylarginine deiminase 4. Source data

Knowledge of self-antigen reactivities of regulatory T cells (Treg) is an important aspect of understanding how they suppress inflammation. To determine whether TCR-MAP could deconvolute class II-restricted Treg specificities, we performed mouse peptidome screens using a Treg TCR (clone MNO) that recognizes the PAD4_92__–__105_ epitope, VRVSYYGPKTSPVQ (Fig. 2f)^30^. G_5_-targets were transduced with H2-IA^b^ and an invariant chain fused mouse peptidome library, cocultured with mouse MNO TCR^+^ SrtA-Jurkat reporters and screened. The top-scoring hit from the class II mouse peptidome screen was a 56-aa fragment spanning positions 74–103 of PAD4 (Extended Data Fig. 5a). These data support the ability of TCR-MAP to discover self-antigen targets of class I-restricted and class II-restricted mouse TCRs.

Immune-related adverse events (irAEs) that arise in response to immune checkpoint inhibitors pose challenges for many patients during cancer treatment^40^. Myocarditis is a rare but deadly form of irAE where the pathogenesis is driven by clonally expanded CD8^+^ T cells that infiltrate the heart^41,42^. Although TCR reactivities to heart-specific proteins such as ɑ-myosin have been reported, the antigenic source for other dominant T cell clonotypes present in cardiac and skeletal muscle during a mouse model of checkpoint-induced myocarditis remain unknown^42^. To rapidly identify pathogenic myocarditis T cell reactivities, we pooled SrtA-Jurkat reporter cells that expressed five different highly expanded TCRs whose relevance, if any, to the myocarditis was not known and screened them against mouse peptidome-positive H2-K^b^/D^b+^ G_5_-targets (Fig. 5e). From the screen, two 56-aa fragments scored and subsequent validation experiments helped to deconvolute the reactivities to a single TCR clonotype, TCR4 (Fig. 5f). NetMHC analysis of the top-scoring tiles identified several predicted peptide binders for creatine kinase S type (CKMT2) and RIKEN cDNA on H2-K^b^ and H2-D^b+^ (Fig. 5g). TCR4 showed reactivity to three of the eight peptides, where a common motif sequence, XXVRXPKL, was present in the validated epitopes (Fig. 5g). When we looked at the tissue distribution of CKMT2, we found high expression of the gene exclusively in the heart (Fig. 5h)^43^. The reactivity of TCR4 against CKMT2 strongly suggests that this clonotype contributes to the pathology of myocarditis through recognition of self-antigens expressed in cardiac tissue. Through TCR multiplexing, we demonstrate that TCR-MAP can quickly identify new reactivities of TCRs of unknown etiology and provide detail to better understand pathological mechanism.

### TCR-MAP predicts adverse cross-reactivities of clinical TCRs

Adoptive T cell therapy is a promising clinical strategy to eliminate tumors^44,45^. One approach within the cell therapy space has been to engineer high-affinity TCRs targeting self-antigens expressed by cancer cells^46,47^. While TCRs can be engineered to improve antigen recognition, undesirable on-tumor and off-tumor reactivities have been observed upon such affinity enhancement strategies, leading to complications during treatment^48–50^. In one example, TCR engineering to enhance recognition of the HLA-A*01:01-restricted melanoma-associated antigen A3 (MAGEA3) showed promising ex vivo efficacy for several affinity-enhanced TCRs tested^51^. However, one of the affinity-enhanced clonotypes, the a3a TCR, was found to cross-react with a peptide from the cardiac protein titin (TTN) leading to cardiogenic shock and death of two patients during treatment for melanoma^51–53^.Therefore, there is a need to not only comprehensively map candidate TCRs for reactivities against self-targets that may be of clinical benefit, but also screen against off-target antigens that may yield adverse events prior to clinical use.

**Fig. 6:**
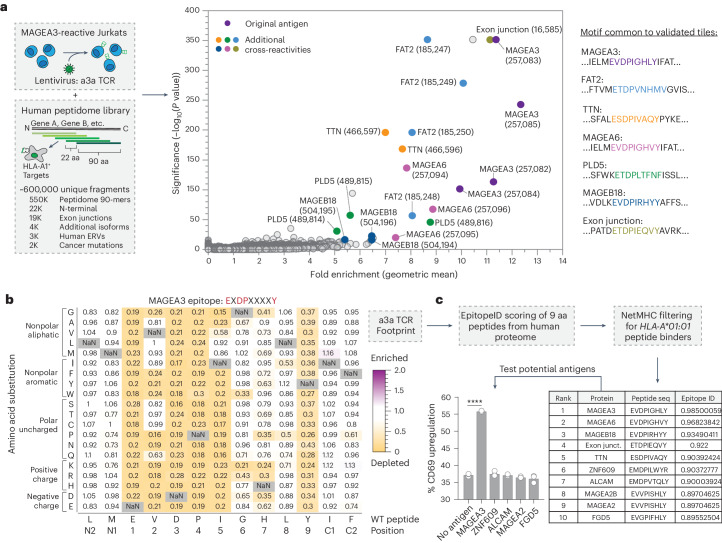
Therapeutic application of TCR-MAP to predict adverse cross-reactivities of clinical TCR. **a**, Schematic of the human genome-wide TCR-MAP screen of the a3a TCR^+^ SrtA-Jurkats and peptide enrichment results. The 90-aa peptides tiling across the human proteome with 22-aa overlap were expressed in HLA-A*01:01^+^ G_5_-target cells. These cells were screened with the evolved MAGEA3 TCR. Each dot represents one peptide, with the *y* axis plotting the −log_10_ adjusted *P* values determined by Mageck and the *x* axis indicating the geometric mean of the enrichment of the peptide across three replicates. Fold enrichment is defined as the ratio of the abundance of the peptide in the sorted population relative to the input library. Peptides highlighted in red contain the epitope from MAGEA3. The remaining colored dots indicate additional cross-reactive fragments that were validated. Validated peptides that scored in the a3a TCR screen are listed with the antigenic epitope colored. **b**, Saturation mutagenesis screen heatmap representing the relative recognition of mutant epitopes from MAGEA3 relative to the original sequence. Each box in the heatmap shows the relative enrichment or depletion of a single mutant peptide, where the amino acid along the *x* axis was substituted to the amino acid indicated along the *y* axis. **c**, EpitopeID ranking of potentially cross-reactive peptides. Saturation mutagenesis heatmap values were used to rank 9-aa peptides tiling the entire human proteome. Scored peptides were then filtered for HLA-A*01:01 binders by NetMHC and listed in descending order of the calculated stimulation potential score. Peptides 6–10 were tested for reactivity by performing peptide pulsing experiments in HLA-A*01:01^+^ G_5_-target cells and the CD69 upregulation of a3a SrtA-Jurkats is reported. ****P < 0.0001 for each group relative to the no-antigen control, determined by a two-tailed *t*-test. Each dot represents a different biological replicate, where error bars indicate the mean and s.d. Peptide pulsing experiments are representative of *n* = 3 independent biological replicates. Source data

On the basis of TCR-MAP to accurately define cross-reactive self-antigens for a given TCR (Fig. 4), we sought to test whether we could comprehensively map the autoreactive landscape of the a3a TCR. We performed human peptidome screens by culturing HLA-A*01:01^+^ G_5_-target cells with SrtA-Jurkat cells transduced with a3a TCR and sorted the top 1% of biotinylated G_5_-target cells to characterize the full self-reactivity profile of the a3a TCR (Fig. 6a). Among the top enriched hits from the screen were previously detected antigens for the pre-enhanced TCR including MAGEA3, MAGEA6, FAT2 (a protocadherin) and PLD5 (a phospholipase) (Fig. 6a)^13^. Several overlapping fragments from TTN showed significant enrichment, as did previously uncharacterized reactivities to MAGEB18 and a predicted exon splice junction in the calcium-responsive transcription factor gene (Fig. 6a). Close inspection of the enriched peptide tile sequences from the screen uncovered the critical EXDPXXXY motif that is likely recognized by the a3a TCR^51^ (Fig. 6a).

### EpitopeID detection of cross-reactive peptides

To further examine whether all possible cross-reactivities of the a3a TCR were discovered, we performed saturation mutagenesis screens to generate a high-resolution TCR footprint (Fig. 6b). Using the saturation mutagenesis enrichment scores, we developed an algorithm called EpitopeID that predicts a rank order of peptides in the human proteome that may be recognized by the a3a TCR (Methods). EpitopeID leverages the fold enrichment values to generate a scoring matrix based on the relative reactivity of a given peptide variant and performs in silico analysis to score the relative stimulatory potential of 9-mer peptides derived from the human proteome (Fig. 6c). Further filtering of potential antigens for HLA-A*01:01 binders was conducted using NetMHC^38^. From this analysis, the top five predicted peptides were derived from the proteins that scored in the human peptidome screens. Two other peptides that weakly scored in the screen were in the top 65 predicted off-targets, PLD5 (44) and FAT2 (64). In addition, our analysis uncovered four peptides from ZNF609, ALCAM, MAGEA2 and FGD5 that scored highly but were not hits in the human peptidome screen. We synthesized peptides for each of the untested epitopes and performed peptide pulsing validation experiments. Only the positive control peptide derived from MAGEA3, EVDPIGHLY, was able to stimulate the a3a TCR^+^ Jurkat cells (Fig. 6c), suggesting that the screen recovered the majority of cross-reactive antigens from the human peptidome. Thus, TCR-MAP has utility for preclinical studies where reducing the risk of engineered TCRs is a primary goal.

## Discussion

Here we characterized an antigen discovery method called TCR-MAP that uses a TCR-stimulated circuit in immortalized T cells to activate sortase-mediated tagging of engineered APCs expressing genetically encoded peptides on MHCs of interest. We demonstrated that TCR-MAP accurately captures self-reactivities or viral reactivities with high throughput and sensitivity for a diverse set of MHC class I-restricted (CD8^+^) and class II-restricted (CD4^+^) T cells and for TCRs derived from humans or mice.

TCR-MAP has several advantages over previously reported antigen discovery methods to facilitate large-scale T cell antigen discovery efforts. Firstly, the reagents and tools used to perform TCR-MAP are readily available in all labs that do mammalian cell culture and do not require specialized protocols such as barcoded tetramer generation or equipment such as microfluidic devices^9,54–56^. Secondly, the system uses target cells that can process long polypeptides or full-length proteins expressed in the cytosol or MHC class II loading compartments for presentation on many different MHC class I or class II molecules, respectively. This negates a priori knowledge of antigenic epitopes and enables synthesis of highly customizable and scalable genetic libraries where the identity of the antigen is unknown^7,57–59^. Thirdly, TCR-MAP takes advantage of immortalized cell lines for antigen discovery efforts, which avoids donor-to-donor variability associated with using primary T cells for cytotoxicity or activation assays and circumvents the need for obtaining patient T cells for antigen discovery or subsequent validation efforts^8,12,13,15^. In addition, engineering of immortalized cell lines allows for species-agnostic T cell antigen discovery and we highlighted here the ability of the method to accurately identify cognate TCR reactivities for both humans and mice. Fourthly, the reporter of cognate T cell reactivities is the biotinylation of G_5_-target cells, whereby the signal is quantitative and proportional to the strength of the antigen recognition and avoids cytotoxic effects on the target cells^12,13,15^. This opens the possibility of enriching live target cells and growing them for further signal amplification through several rounds of enrichment and subsequent rescreening. Thus, these last two points highlight some of the strengths inherent in TCR-MAP and outline the design advancements that were incorporated in our second-generation T cell antigen discovery method, building on our previous T-Scan technologies. Lastly, we demonstrated that the method can accurately identify cognate antigens from genetic screens where single or pooled TCRs are used. Moreover, detailed TCR binding footprints can be generated and used to assess potential cross-reactivities. TCR-MAP is, thus, a sensitive and convenient antigen discovery method to deconvolute TCR specificities at scale and has broad application across species.

While powerful, there are several aspects of T cell antigen discovery that the current design of TCR-MAP does not address. Firstly, although we demonstrated that TCR-MAP can accurately capture low-affinity and high-affinity antigens when several TCRs are combined, larger-scale antigen screening efforts that combine multiple TCR clonotypes with varying antigen affinities would benefit from further optimization of the effector-to-target cell ratios used in a screening context and the maximal number of clonotypes to pool on the basis of known cognate TCRs as internal controls. Secondly, without further engineering of target cells, TCR-MAP is unlikely to capture T cell reactivities to post-translationally modified (PTM) peptides. Future work expressing enzymes that catalyze modifications such as phosphorylation (by kinases) or citrullination (by peptidyl arginine deiminases) in target cells may enable PTM antigen discovery efforts. Lastly, we did not demonstrate the ability of TCR-MAP to deconvolute T cell reactivities against nonpeptide antigens such as lipids (for CD1d-restricted invariant natural killer T cells) or metabolites (for MR1-restricted mucosal-associated invariant T cells). Owing to the flexibility of the TCR-MAP method, however, additional engineering of target cells to express the restricting MHC allele of interest coupled with genetic screening strategies to activate lipid or vitamin B metabolic pathways may expand the possibility of unbiased T cell antigen discovery for T cell subsets not restricted by MHC classes I and II.

There is a growing need to understand the pathological mechanisms that drive irAEs in patients who receive immune checkpoint blockade treatment. Using multiplexed TCR-MAP screens against our mouse proteome library, we were able to rapidly identify cardiac self-reactivity against CKMT2 for an expanded TCR clonotype derived from a mouse model of myocarditis. It will be interesting to assess whether CKMT2 also serves as an autoantigen for expanded CD8 TCRs in human patients with myocarditis. Thus, TCR-MAP holds promise as a tool for T cell antigen discovery efforts in mouse models, which can then inform new insights and generate hypotheses for evaluation in human disease.

The clonal selection theory proposes that each individual lymphocyte bears a single type of receptor with unique specificity^2^. However, we now appreciate that TCRs can exhibit some level of cross-reactivity and cross-restriction, thereby increasing the total number of foreign antigens that T cells can respond to^2,32–34,60^. As adoptive T cell therapies gain ground as a cancer treatment option, there is a growing need to test the safety profile of clinical TCRs before infusion into patients. By vetting the a3a MAGEA3*-*specific TCR using TCR-MAP, we discovered several cross-reactive antigens that were unknown at the time of clinical use. Knowledge of the a3a MAGEA3 TCR specificity against self-proteins such as TTN may have motivated greater precaution and further engineering before patient treatment that could have saved lives. Systematic characterization of T cell reactivity profiles using comprehensive antigen mapping technologies such as TCR-MAP will undoubtedly help to reduce the risk of therapeutic TCRs moving forward.

Lastly, we demonstrated that the EpitopeID algorithm has utility in predicting off-target effects of TCR specificity. Many approaches have been proposed to predict the epitope specificity of TCRs^61^. Computational approaches for these predictions commonly use receptor–peptide interaction databases such as VDJdb, IEDB and PIRD and/or additional information such as from CDR3 sequencing. In contrast, EpitopeID is a functional readout of the saturation mutagenesis TCR footprint using endogenous antigen presentation, which provides an empirical, high-resolution starting point from which cross-reactivity predictions can be made. Furthermore, by querying TCR footprints against a proteome of interest rather than against all possible *k*-mers, we cast a wide net in terms of search space for potential cross-reactors while also imposing a logical constraint to sequences that are likely to be observed. In our study, TCR-MAP saturation mutagenesis footprinting, coupled with EpitopeID, predicted the top five scoring peptides in the human peptidome screen itself. In addition, it predicted two additional cross-reactive epitopes, from PLD5 and FAT2, within the top 65 predictions. One explanation for why these cross-reactivities did not receive a higher score is that the algorithm was trained on the effects of single-amino acid changes. Therefore, it is possible that changes owing to pairs of amino acids can act in a cooperative, nonlinear fashion to influence the TCR loop interaction structure in currently unpredictable ways^62^. Future efforts that use mutagenesis matrices that contain all possible double-amino acid substitutions might, thus, further improve TCR reactivity predictions^8,13,47^.

## Methods

### Cell culture

HEK-293T (CRL-3216) and TCRβ-null Jurkat (J.RT3-T3.5) cell lines were obtained from the American Type Culture Collection (ATCC). HEK-293T cells were cultured in DMEM (Gibco, 11995065) with 10% FBS (HyClone) and 1% penicillin–streptomycin (Invitrogen, 15140-122). TCRβ-null Jurkat cells were cultured in RPMI (Gibco, A10491-01) with 10% FBS (HyClone) and 1% penicillin–streptomycin (Invitrogen, 15140-122). All cell lines were regularly tested for mycoplasma and were all negative. Cells were obtained directly from ATCC and, thus, were not authenticated.

### Generation of TCR-MAP target cell lines

HEK-293T cells were transfected with sgRNAs targeting conserved sequences across the HLA-A, HLA-B and HLA-C locus to generate HLA class I-null target cells as described previously^13^. To generate target cells for MHC class II antigen presentation, HEK-293T cells were first transduced with a lentiviral vector containing an EF1α promoter driving expression of CIITA (UniProt P33076) and CTSS (UniProt P25774). Cells were sorted for high HLA II expression. Cas9 protein (Thermo Fisher Scientific, A36499) was complexed with 900 pmol of sgRNAs targeting HLA-DPB1, HLA-DPA1, HLA-DQB1, HLA-DQA1, HLA-DRA, HLA-DRB1 and HLA-DRB5 (Invitrogen TrueGuide Synthetic gRNAs, A35510) in Opti-MEM medium and incubated for 5 min at room temperature to form Cas9 ribonucleoproteins and added to cells (Thermo Fisher Scientific, Lipofectamine CRISPRMAX Cas9 Transfection Reagent, CMAX00003). After incubation for 48 h, the cells were assessed for HLA II expression and cells exhibiting diminished cell-surface HLA class II molecules were then single-cell cloned by sorting into 96-well plates. Both the HLA class I-null and HLA class I/II-null HEK-293T cell lines were transduced with lentivirus containing EF1α-G_5_-mCD40-neomycin constructs. HLA-A*02:01, HLA-A*01:01, HLA-DRB1*11:01, HLA-DRB1*15:01, HLA-DRB1*04:01, HLA-DRB1*01:02, HLA-DRA*01:01, HLA-DPB1*04:01;DPA*01:03, HLA-DPB1*04:02;DPA*01:03, HLA-DQB1*03:01;DQA*03:01 and HLA-DQB1*05:01;DQA*01:01 sequences were obtained from the IPD-IMGT/HLA database^63^ and synthesized as gBlocks (IDT). Mouse MHC allele sequences were obtained from UniProt and synthesized as gBlocks (IDT): H2-K^b^ (UniProt P01901), H2-L^d^ (UniProt P01897), H2-IA^d^ (UniProt P01921) and H2-IA^b^ (UniProt P14483). Human and mouse MHC alleles were cloned into pDONR221. For expression, they were Gateway-cloned into pHAGE-EF1α-DEST expression vectors with variable antibiotic selection and fluorophore markers.

### Generation of TCR-MAP Jurkat cell lines

TCRβ-null Jurkat cells were spinfected with lentivirus at varying concentrations to achieve a multiplicity of infection (MOI) < 1 and introduced with CD4 or CD8 coreceptors, the NFAT-SrtA reporter and TCRs of interest. A total of 1 × 10^6^ cells were spun with 8 μg ml^−1^ polybrene (Millipore, TR-1003-G) and lentivirus for 30 min at 800*g* in 12-well plates. Cells were incubated at 37 °C and the virus was washed off after 24 h. After spinfection for 48 h, the cell-surface expression of constructs was tested by flow cytometry. Human CD4 (UniProt P01730) and CD8 (P01732 and P10966) and mouse CD8 (UniProt P01731 and P10300) coreceptors were synthesized as gBlocks (IDT) and cloned into pDONR221 (Gateway 12536017). CD8β and CD8α receptors were separated by porcine teschovirus 1 (P2A)^64^. The CD4 coreceptor used for mouse class II antigen discovery was engineered by fusing the mouse extracellular domain of CD4 (UniProt P06332) with the human transmembrane and cytoplasmic tail of CD4. Codon-optimized TCRβ and TCRα variable sequences (V-CDR3-J domains) of TCRs used in the study were fused to mouse TCR constant regions, gene-synthesized (Twist Biosciences) and subcloned in pHAGE-EF1α-DEST-PGK-Bsd expression vectors. Affinity-matured a3a MAGEA3 TCRs were designed using human TCR constant regions and cloned into pHAGE-EF1α-DEST-PGK-ZsGreen vectors for expression. Each TCR was encoded a single construct containing TCRα P2A TCRβ with either the mouse or human TCR constant region.

### Generation of TCR-MAP reporter constructs

Plasmids encoding G_5_–Myc–mCD40 and Flag–SrtA–mCD40L were kindly provided by Gabriel Victora^17^. G_5_–Myc–mCD40 was subsequently cloned into the pHAGE-EF1α-DEST-PGK-neomycin vector. Flag–SrtA–mCD40L was cloned into inducible expression vectors containing leucine zipper (ZIP) domains and NFAT response elements in various combinations upstream of a minimal interleukin 2 (IL-2) promoter (Extended Data Fig. 1a).

### Lentiviral production

HEK-293T cells were transfected with second-generation lentiviral packaging plasmids pMD2.G (Addgene, cat. no. 12259) and psPAX2 (Addgene, cat. no. 12260), encoding VSV-G, Tat, Rev and Gag-Pol. Transfection was performed using PolyJet In Vitro DNA Transfection Reagent (SignaGen, SL100688) according to the manufacturer’s protocol. Viral supernatants were collected 48 and 72 h after transfection, passaged through a 0.45-µm filter and added to cells.

### SrtA substrates

Biotin–aminohexanoic acid–LPETGS (C-terminal amide, 95% purity) was purchased from LifeTein (custom synthesis) and stock solutions were prepared in PBS at 20 mM as previously reported^17^.

### Peptide pulsing and endogenous antigen expression cocultures

Small-scale validation experiments testing TCR reactivities against various antigens were performed in 96-well plates adding 100,000 G_5_-target cells with 100,000 SrtA-Jurkat cells in the presence of 50 μM of LPETG–biotin substrate. After 6–16-h incubation at 37 °C, cells were washed twice with PBS supplemented with 0.5% BSA and 2 mM phosphate-buffered EDTA (PBE) to remove excess LPETG–biotin before analysis by flow cytometry. Peptides (GenScript custom peptide synthesis) used for peptide pulsing experiments were added to G_5_-target cells at a concentration of 1 μM for 1 h before cells were washed and cultured with SrtA-Jurkat cells. For endogenous expression of antigens, 56-mer or 90-mer peptide fragments were reverse-translated and synthesized as gBlocks (IDT) with 5′ and 3′ BP recombination sites. Peptide fragments were Gateway-cloned into pHAGE-CMV-Nflag-HA-DEST-IRES-Puro or pHAGE-CMV-CD74_1__–__80_-DEST-PGK-Puro expression vectors for class I or class II antigen presentation experiments, respectively. Peptides and antigens used for TCR3898-2 validation are reported in Extended Data Fig. 4. All other peptides and antigens expressed in target cells were as follows:

Pp65 56-mer: RLKAESTVAPEEDTDEDSDNEIHNPAVFTWPPWQAGILARNLVPMVATVQSGARA*

CTAG1B 90-mer: APPLPVPGVLLKEFTVSGNILTIRLTAADHRQLQLSISSCLQQLSLLMWITQCFLPVFLAQPPSGQRR*

HIV gag 56-mer: SILDIRQGPKEPFRDYVDRFYKTLRAEQASQEVKNWMTETLLVQNANPDCKTILKA

OT-II OVA 90-mer: LMAMGITDVFSSSANLSGISSAESLKISQAVHAAHAEINEAGREVVGSAEAGVDAASVSEEFRADHPFLFCIKHIATNAVLFFGRCVSP*

PAD4 56-mer: EVTLQVKAASSRTDDEKVRVSYYGPKTSPVQALIYITGVELSLSADVTRTGRVKPA

OGDH 56-mer: EEEVAITRIEQLSPFPFDLLLKEAQKYPNAELAWCQEEHKNQGYYDYVKPRLRTTI

SLC31A1 56-mer: HSHGGGDSMMMMPMTFYFDFKNVNLLFSGLVINTPGEMAGAFVAVFLLAMFYEGLK

SIINFEKL (WT 8-mer): SIINFEKL. Variants of SIINFEKL used contained the indicated substitution from the WT sequence.

OT-II peptide: KISQAVHAAHAEINEAG

PAD4 peptide: VRVSYYGPKTSPVQ

SLC31A1 predicted peptide binders: PMTFYFDF, MPMTFYFDF and MMMPMTFYF

MAGEA3 peptide: EVDPIGHLY

ZNF609 peptide: EMDPILWYR

ALCAM peptide: EMDPVTQLY

MAAGEA2B peptide: EVVPISHLY

MAGEA2 peptide: EVVPISHLY

FGD5 peptide: EVGPIFHLY

### Flow cytometry

Cells were stained for at least 30 min in PBE with antibodies and then washed two times in PBE. Samples were acquired using LSR-II (BD Biosciences) or CytoFLEX (Beckman Coulter) flow cytometers and data were analyzed using FlowJo (version 10.8.2) software. All antibodies or cell-surface staining reagents were from BioLegend and were used at 0.5−1 μl per million cells (APC anti-mouse CD40, clone 3/23; BV421 streptavidin, 405226; PE or BV421 anti-human CD69, clone FN50; APC anti-mouse CD154 (CD40L), clone SA047C3; PE anti-biotin, 1D4-C5; APC anti-human HLA-A, HLA-B and HLA-C, clone W6/32; APC anti-human HLA-DR, HLA-DP and HLA-DQ, clone Tu39; BV421 anti-mouse MHC class II, clone M5/11.15.2; PE anti-human CD4, clone RPA-T4; FITC anti-mouse CD4, clone RM4-5; BV785 anti-human CD8, clone SK1; BV421 anti-mouse CD8, clone 53-6.7; APC anti-mouse H2Kb, clone AF6-88.5).

### Fluorescence-activated cell sorting-based TCR-MAP screens

For TCR-MAP screens, 35 μl of antibody (anti-mouse CD40, clone 3/23 and BV421 streptavidin) in a total volume of 1 ml was used per 80 million cells. Staining was conducted for 30 min at 4 °C; cells were then washed in PBE before sorting. Sorting was performed on a Sony MA900 instrument where the top 1% of biotinylated target cells were isolated.

### Magnetic bead enrichment-based TCR-MAP screens

CMV-specific TCR-MAP screens were performed by culturing HLA-A*02:01^+^ G_5_-target cells with NLV3 TCR^+^ SrtA-Jurkats at a ratio of 1:1 for 8–12 h in the presence of 50 μM LPETG–biotin. Screens were performed at 1,000× library representation with three biological replicates. Cells were collected using enzyme-free dissociation medium (PBS supplemented with 2 mM EDTA) and washed three times in PBE before performing streptavidin microbead magnetic column isolation according to the manufacturer’s protocol (Miltenyi Biotec, 130-048-101). Bound cells were eluted and plated in 10-cm plates and grown for 1 week, after which further cocultures and enrichments were performed. Isolated target cells were saved after each round of enrichment for genomic DNA (gDNA) isolation and library preparation for sequencing.

### CMV-specific and virome-wide peptidome libraries

The CMV-focused and virome-wide library was described previously^13,65^. The library was cloned into the pHAGE-CMV-Nflag-HA-DEST-IRES-Puro lentiviral vector by Gateway cloning. This vector enables peptide fragments to be uniformly expressed by identical start codons followed by an N-terminal Flag and HA tag. At least 100× library representation was maintained at each step of cloning. HLA-A*02:01^+^ G_5_-target cells were infected with lentivirus containing the CMV-focused or virome-wide library at an MOI of 3–5 to achieve 1,000× library representation and selected with 1 μg ml^−1^ puromycin for 3 days before use in TCR-MAP screens.

### Saturation mutagenesis library

The SIINFEKL epitope was mutagenized in the context of a 56-mer (LPFASGTMSMLVLLPDEVSGLEQLE**SIINFEKL**TEWTSSNVMEERKIKVYLPRMKME) and an 8-mer (SIINKEKL). Each amino acid in the SIINFEKL epitope (bold) was substituted to each of the other 19 amino acids. As a control, the two adjacent amino acids outside the NLV epitope in the 56-aa version (underlined) were also substituted to each of the other 19 amino acids. This set contained 228 mutants and two WT epitope controls. For the MAGEA3 saturation mutagenesis library, the MAGEA3 epitope was mutagenized in the context of a 56-mer (VIFSKASSSLQLVFGIELM**EVDPIGHLY**IFATCLGLSYDGLLGDNQIMPKAGLLIIV) and a 9-mer (EVDPIGHLY). Each amino acid in the MAGEA3 epitope (bold) was substituted to each of the other 19 amino acids. As a control, the two adjacent amino acids outside the MAGEA3 epitope in the 56-aa version (underlined) were also substituted to each of the other 19 amino acids. These 247 mutants were combined with the two WT epitopes. Each peptide was reverse-translated with nonrare human codons in two different nucleic acid sequences for a total of 460 and 498 oligo sequences. The SIINFEKL and MAGEA3 mutagenesis libraries were combined with 1,034 unrelated peptides (saturation mutagenesis tiles for the BMLF epitope derived from Epstein–Barr virus (EBV) and the MLANA cancer/testis antigen epitope) for a total library size of 1,992 oligo tiles. The 5′ (ACCCGTCACCGGCCA) and 3′ adaptors (GGGCTCGCCACGTCG) were added and oligonucleotides were synthesized by Twist Bioscience. The library was PCR-amplified using primers complementary to the adaptor sequences with overhangs encoding BP recombination sites. The amplified library was cloned into the pDONR221 vector using BP Clonase (Thermo Fisher Scientific) and Gateway-cloned into the pHAGE-CMV-Nflag-HA-DEST-IRES-Puro lentiviral expression vector. At least 100× library representation was maintained during all cloning steps. H2-K^b^^+^ or HLA-A*01:01 G_5_-target cells were infected with lentivirus containing a saturation mutagenesis library MOI of <1 to achieve 1,000× library representation and selected with 1 μg ml^−1^ puromycin for 3 days before use in TCR-MAP screens to test the OT-I or a3a TCRs, respectively.

### Human 90-mer peptidome library

The human peptidome 90-mer library consisted of 90-aa tiles with 22-aa offset covering the entire human proteome, as previously described^15^. In addition, GENCODE protein-coding transcripts (version 29) were subjected to the basic local alignment search tool (BLAST) against the ORFeome library; of the 500 nonredundant ORFs that remained, 90-aa tiles offset by 22-aa were generated. Additional 90-aa tiles offset by 22-aa were generated from ORFs encoded by endogenous retroviral elements (ERVs) from the GEVE database, recurrent cancer mutations from the COSMIC database and hotspot mutations^66^. Aspartate residues were placed before the beginning of each methionine of each 29-aa N-terminal peptide and concatenated with two other N-terminal fragments to generate additional representation of the N terminus of human ORFs. The 5′ (GGAATTCCGCTGCGT) and 3′ adaptors (CAGGGAAGAGCTCGA) were added and oligonucleotides were synthesized by Twist Bioscience. The library was PCR-amplified using primers complementary to the adaptor sequences with overhangs encoding BP recombination sites. The amplified library was cloned into the pDONR221 vector using BP Clonase (Thermo Fisher Scientific) and Gateway-cloned into the pHAGE-CMV-Nflag-HA-DEST-IRES-Puro or pHAGE-CD74_1–80_-DEST-PGK-Puro lentiviral expression vectors for class I or class II antigen discovery efforts, respectively. At least 100× library representation was maintained during all cloning steps. HLA-A*02:01, HLA-A*01:01 or DR^+^DP^+^DQ^+^ G_5_-target cells were infected with lentivirus containing a saturation mutagenesis library MOI of 3–5 to achieve 1,000× library representation and selected with 1 μg ml^−1^ puromycin for 3 days before use in TCR-MAP screens to test the IG4, 3898-2 or a3a TCRs, respectively.

### Mouse 56-mer peptidome library screens

Proteins used for the mouse 56-mer peptidome library were obtained from the following UniProt proteomes: UP000000589 (C57BL/6J), UP000002494 (Brown Norway), UP000002474 (LCMV Armstrong), UP000008479 (Murine polyomavirus A2), UP000158963 (*Mus musculus* polyomavirus 2) and UP000129308 (*Mus musculus* papillomavirus type 1). Peptidome tiles were generated by randomly sampling from the de Bruijn graph representation of the mouse proteome dataset to try to achieve the most uniform distribution of *k*-mers possible. For each protein, additional C-terminal 56-mer tiles were included. Peptide fragments were reverse-translated, adaptors were appended to the 5′ (AGGAATTCCGCTGCGT) and 3′ (ATGGTCACAGCTGTGC) ends and oligonucleotides were synthesized by Twist Bioscience. The library was PCR-amplified using primers complementary to the adaptor sequences with overhangs encoding BP recombination sites. The amplified library was cloned into the pDONR221 vector using BP Clonase (Thermo Fisher Scientific) and Gateway-cloned into the pHAGE-CMV-Nflag-HA-DEST-IRES-Puro or pHAGE-CD74_1–80_-DEST-PGK-Puro lentiviral expression vectors for mouse class I or class II antigen discovery efforts, respectively. At least 100× library representation was maintained during all cloning steps. H2-K^b^^+^, H2-L^d^^+^ or H2-IA^b^^+^ G_5_-target cells were infected with lentivirus containing a saturation mutagenesis library MOI of 3–5 to achieve 1,000× library representation and selected with 1 μg ml^−1^ puromycin for 3 days before use in TCR-MAP screens to test the myocarditis, 2C or 6MNO TCRs, respectively.

### Library preparation for sequencing

gDNA was extracted from sorted or magnetic enrichment-purified cells using the GeneJET Genomic DNA Purification Kit (Thermo Fisher Scientific). Input library cells with at least 40× representation were collected and prepared for each screen. Antigen libraries were prepared for Illumina sequencing following a previously published protocol^13,15^. Samples were sequenced on Illumina MiSeq or NextSeq using the standard Illumina sequencing primers.

### Saturation mutagenesis scoring matrix analysis

For analysis of the saturation mutagenesis footprints, we developed an algorithm called EpitopeID^67^. Overall, the effect of EpitopeID is that the data from a position specific scoring matrix (PSSM), such as that generated by a saturation mutagenesis screen, can be used to compute a numeric score for any sequence of interest, where the numeric score serves as a prediction for how that queried sequence would perform if screened. For a motif length of *N*, with each residue being substituted to the 19 other amino acids, a PSSM of size 20 × *N* would be generated. This results in the value in each cell of this PSSM representing the measured TCR reactivity relative to WT. For any amino acid sequence of length *N*, the score *S* would be computed by the following equation:

$$S=\mathop{\sum }\limits_{i}^{20}\mathop{\sum }\limits_{j}^{N}{s}_{{ij}}$$

where *i* represents the identity of the amino acid, *j* represents the position in the queried amino acid sequence and *s*_*ij*_ represents the saturation mutagenesis screen score relative to WT for the peptide with substitution *i* at position *j.* Furthermore, *p*_*j*_ represents the ‘position weight’ of position *j* in the epitope and *a*_*i*_ represents the ‘residue weight’ for substituting amino acid *i* at position *j*. Screen score *s*_*ij*_ ranges from 0 to 1, with 0 indicating no TCR activation and 1 indicating activation greater than or equal to WT peptide. To define the amino acid interval that contains an epitope, we compute ${q}_{j}=\frac{{-s}_{{{\rm{WT}}{j}}}\,+\,{\sum }_{i}^{20}\left(1-{s}_{{ij}}\right)}{20-1}$ for a given position *j*, which can be thought of as the average decrease in activation observed by substituting the residue at position *j* of the epitope to one of the 19 other amino acids. Peptide positions at which there is a large decrease in activation upon substitution are likely to be critical residues for the TCR–peptide interaction. The amino acid interval containing the epitope of interest was defined as the continuous range containing all positions with *q*_*j*_ ≥ 0.5 × max(*q*_1_, …, *q*_*N*_). Amino acid positions outside of the interval containing the epitope were given a position weight of *pj* = 0. Amino acid positions inside the interval containing the epitope were given a position weight of ${p}_{j}=\frac{{q}_{j}}{\max \left({q}_{1},\ldots, {q}_{N}\right)}$. The implementation of EpitopeID in this paper used a uniform amino acid weight for all substitutions across all positions; however, the algorithm is capable of accounting for a customized amino acid weight matrix. To discover potential TCR cross-reactivities, human ORFs from our genome-wide human peptidome library were computationally tiled using a window size of *N* and an increment of one amino acid between tiles to represent all available *k*-mers of length *N*. Each *N*-mer was then scored using the above equation to assess its potential similarity to the motif tested by saturation mutagenesis. For each human *N*-mer sequence, this score *S* was normalized by the theoretical maximum for that PSSM and reported as a percentage. Human peptides containing the best-scoring *N*-mers from this analysis were then considered for subsequent validation.

### Statistical analysis

Statistical tests were conducted using Prism (GraphPad version 9.0) software. Unpaired, two-tailed Student’s *t*-tests and one-way analyses of variance (ANOVAs) with Tukey–Kramer multiple-comparison tests to further examine pairwise differences were used. The statistical analyses performed for the various experiments are outlined in the figure legends.

### Sequence alignment and analysis

Read processing and alignment were performed with Cutadapt^68^ and Bowtie 2 (ref. ^69^), respectively. The fractional abundance of each antigen in each screen replicate was divided by the fractional abundance in the presort input library to calculate the fold enrichment of the peptide tile. Mageck version 0.5.8 was used to assign *P* values to peptide fragments in TCR-MAP screens, whereby different codons used for a peptide were treated as the sgRNAs and amino acid sequences were used as the genes. Screen figures were generated using DataGraph (version 4.7).

### Reporting summary

Further information on research design is available in the Nature Portfolio Reporting Summary linked to this article.

## Online content

Any methods, additional references, Nature Portfolio reporting summaries, source data, extended data, supplementary information, acknowledgements, peer review information; details of author contributions and competing interests; and statements of data and code availability are available at 10.1038/s41587-024-02248-6.

## Supplementary information

Reporting Summary

Supplementary Note 1 MTA template.

## Source data

Source Data Fig. 3b Peptide tiles enriched in the NLV3 TCR viral peptidome screen.

Source Data Fig. 3d Results of the SIINFEKL saturation mutagenesis footprint screens with the OT-I TCR.

Source Data Fig. 4a Peptide tiles enriched in the IG4 TCR human peptidome screen.

Source Data Fig. 4c Peptide tiles enriched in the pooled IG4, DMF5 and three irrelevant A2 restricted TCR screens against the human peptidome.

Source Data Fig. 5a Peptide tiles enriched in the 2C TCR mouse peptidome screen.

Source Data Fig. 5e Peptide tiles enriched in the pooled myocarditis TCR screen.

Source Data Fig. 6a Peptide tiles enriched in the MAGEA3 a3a TCR human peptidome screen.

Source Data Fig. 6b,c List of peptides and their EpitopeID scores when analyzed against the 9-aa human peptidome library. Peptides were then filtered for HLA-A*01:01 binders.

Source Data Extended Data Fig. 3 Results of the NLV3 TCR screened against the CMV peptidome.

Source Data Extended Data Fig. 4 Peptide tiles enriched in the TCR3898-2 human peptidome screen.

Source Data Extended Data Fig. 5 Results of the 6MNO TCR screen against the mouse peptidome.

## Data Availability

Plasmids and cell lines generated in this study are available upon reasonable request and are subject to a material transfer agreement (MTA) from the lead contact. The MTA template and conditions for its use are provided as Supplementary Note 1. Normal tissue FPKM data were obtained from The Human Protein Atlas (https://www.proteinatlas.org/about/download). Source data are provided with this paper.

## Code availability

A custom EpitopeID script^67^ to perform saturation mutagenesis scoring matrix analysis is available at 10.5281/zenodo.8103914 (see Methods for details).
