# Supplementary material for: High-throughput discovery of MHC class I- and II-restricted T cell epitopes using synthetic cellular circuits
Source: Nat Biotechnol. 2024 Jul 2;43(4):623–34. doi: 10.1038/s41587-024-02248-6 (PMC11994455; doi:10.1038/s41587-024-02248-6)
Supplement: Supplementary file 1 — Reporting Summary [file 41587_2024_2248_MOESM1_ESM.pdf]

nature portfolio

Corresponding author(s): S. J. E.

Last updated by author(s): Apr 1, 2024

Reporting Summary

Nature Portfolio wishes to improve the reproducibility of the work that we publish. This form provides structure for consistency and transparency in reporting. For further information on Nature Portfolio policies, see our Editorial Policies and the Editorial Policy Checklist.

Statistics

For all statistical analyses, confirm that the following items are present in the figure legend, table legend, main text, or Methods section.

n/a	Confirmed	
☐	☒	The exact sample size (n) for each experimental group/condition, given as a discrete number and unit of measurement
☐	☒	A statement on whether measurements were taken from distinct samples or whether the same sample was measured repeatedly
☐	☒	The statistical test(s) used AND whether they are one- or two-sided<br>*Only common tests should be described solely by name; describe more complex techniques in the Methods section.*
☒	☐	A description of all covariates tested
☐	☒	A description of any assumptions or corrections, such as tests of normality and adjustment for multiple comparisons
☐	☒	A full description of the statistical parameters including central tendency (e.g. means) or other basic estimates (e.g. regression coefficient) AND variation (e.g. standard deviation) or associated estimates of uncertainty (e.g. confidence intervals)
☐	☒	For null hypothesis testing, the test statistic (e.g. F, t, r) with confidence intervals, effect sizes, degrees of freedom and P value noted<br>*Give P values as exact values whenever suitable.*
☒	☐	For Bayesian analysis, information on the choice of priors and Markov chain Monte Carlo settings
☒	☐	For hierarchical and complex designs, identification of the appropriate level for tests and full reporting of outcomes
☒	☐	Estimates of effect sizes (e.g. Cohen's d, Pearson's r), indicating how they were calculated

Our web collection on statistics for biologists contains articles on many of the points above.

Software and code

Policy information about availability of computer code

Data collection	Data collection was performed via FACSDiva 6.0 (BD) and CytoFLEX Acquisition and Analysis Software (version 2.3).
Data analysis	Flow cytometry analysis was performed using FlowJo v10.8.2 (BD).<br>Bar plots and scatter plots were generated using GraphPad Prism 9.<br>Statistical analysis was performed using GraphPad Prism 9 software.<br>Read processing and alignment were performed with CutAdapt66 and Bowtie 267, respectively.<br>Mageck 0.5.8 was used to assign p-values to peptides in TCR-MAP screens.<br>Screen results and saturation mutagenesis footprint figures were generated using DataGraph v4-7.<br>EpitopeID script for saturation mutagenesis scoring matrix analysis is outlined in the Methods section.

For manuscripts utilizing custom algorithms or software that are central to the research but not yet described in published literature, software must be made available to editors and reviewers. We strongly encourage code deposition in a community repository (e.g. GitHub). See the Nature Portfolio guidelines for submitting code & software for further information.

March 2021

Data

Policy information about availability of data

All manuscripts must include a data availability statement. This statement should provide the following information, where applicable:

- Accession codes, unique identifiers, or web links for publicly available datasets
- A description of any restrictions on data availability
- For clinical datasets or third party data, please ensure that the statement adheres to our policy

Plasmids and cell lines generated in this study are available upon reasonable request and are subject to a Materials Transfer Agreement (MTA) from the lead contact. A copy of the MTA and conditions for its use are provided as part of supplemental information of the manuscript. Source data are provided with this paper. Normal tissue FPKM data was obtained from The Human Protein Atlas (https://www.proteinatlas.org/about/download).

Human research participants

Policy information about studies involving human research participants and Sex and Gender in Research.

Reporting on sex and gender	N/A
Population characteristics	N/A
Recruitment	N/A
Ethics oversight	N/A

Note that full information on the approval of the study protocol must also be provided in the manuscript.

Field-specific reporting

Please select the one below that is the best fit for your research. If you are not sure, read the appropriate sections before making your selection.

☒ Life sciences ☐ Behavioural & social sciences ☐ Ecological, evolutionary & environmental sciences

For a reference copy of the document with all sections, see nature.com/documents/nr-reporting-summary-flat.pdf

Life sciences study design

All studies must disclose on these points even when the disclosure is negative.

Sample size	No sample-size calculations were performed. Each experiment was performed at least three times via independent biological experiments with three technical replicates to ensure sample size was sufficient for experimental interpretation. Since the results were consistent between experiments, three was generally deemed sufficient. No data were excluded from the analysis.
Data exclusions	No data were excluded from the analysis.
Replication	All experiments conducted with cell culture samples were repeated at least three times via independent biological experiments. All attempts at replication were successful.
Randomization	No randomization was performed as it is not relevant to the present study as there is no treatment involved. All conditions were assigned in advance by the experimentalist and thus well defined.
Blinding	No blinding was performed because there was no group allocation performed.

Reporting for specific materials, systems and methods

We require information from authors about some types of materials, experimental systems and methods used in many studies. Here, indicate whether each material, system or method listed is relevant to your study. If you are not sure if a list item applies to your research, read the appropriate section before selecting a response.

Materials & experimental systems

n/a	Involved in the study
☐	☒ Antibodies
☐	☒ Eukaryotic cell lines
☒	☐ Palaeontology and archaeology
☒	☐ Animals and other organisms
☒	☐ Clinical data
☒	☐ Dual use research of concern

Methods

n/a	Involved in the study
☒	☐ ChIP-seq
☐	☒ Flow cytometry
☒	☐ MRI-based neuroimaging

Antibodies

Antibodies used	Cells were stained for at least 30min in PBE (1x PBS with 2% BSA and 2mM EDTA) with antibodies, washed two times in PBE. All antibodies or cell-surface staining reagents were from BioLegend and were used at 0.5 -1ul per million cells (APC anti-mouse CD40, clone 3/23; BV421 Streptavidin, 405226; PE or BV421 anti-human CD69, clone FN50; APC anti-mouse CD154 (CD40L), clone SA047C3; PE anti-biotin, 1D4-C5; APC anti-human HLA-A, HLA-B and HLA-C, clone W6/32; APC anti-human HLA-DR, DP, DQ, clone Tu39; BV421 anti-mouse MHC-II, clone M5/11.15.2; PE anti-human CD4, clone RPA-T4; FITC anti-mouse CD4, clone RM4-5; BV785 anti-human CD8, clone SK1; BV421 anti-mouse CD8, clone 53-6.7; APC anti-mouse H2Kb, clone AF6-88.5).
Validation	No explicit antibody validation was performed in this study. However, most antibodies were used in the context of gene knockout or cDNA over-expression and all results were consistent with the antibodies recognizing their intended targets. Validation and other pertinent information to each antibody can be found on the manufacturer's website. Each lot of the antibody used was quality control tested by immunofluorescent staining with flow cytometric analysis by the manufacturer as part of their quality control.

Eukaryotic cell lines

Policy information about cell lines and Sex and Gender in Research

Cell line source(s)	HEK-293T (CRL-3216) and TCRbeta-null Jurkat (J.RT3-T3.5) were obtained from ATCC.
Authentication	None of the cell lines used were authenticated.
Mycoplasma contamination	All lines used for the study tested negative for Mycoplasma contamination.
Commonly misidentified lines (See ICLAC register)	No commonly misidentified cell lines were used.

Flow Cytometry

Plots

Confirm that:

☒ The axis labels state the marker and fluorochrome used (e.g. CD4-FITC).

☒ The axis scales are clearly visible. Include numbers along axes only for bottom left plot of group (a 'group' is an analysis of identical markers).

☒ All plots are contour plots with outliers or pseudocolor plots.

☒ A numerical value for number of cells or percentage (with statistics) is provided.

Methodology

Sample preparation	HEK-293T cells were removed from the plate via incubation at 37C for 1min in 0.25% trypsin. Cells were stained for at least 30 m in PBE with antibodies, washed two times in PBE before flow cytometry analysis or sorting.
Instrument	Sorting was performed on a Sony MA900 instrument. Analysis was performed on an LSR-II (BD) or CytoFLEX (Beckman Coulter).
Software	Data was collected with FACSDiva (BD) and analysis was performed using FlowJo v10.8.2 (BD).
Cell population abundance	The purity of post-sort samples was determined by culturing the cells and staining and analyzing on a flow cytometer. The abundance of each sort varied.
Gating strategy	The gating strategy relevant for all experiments is shown in Fig. 1b.

☒ Tick this box to confirm that a figure exemplifying the gating strategy is provided in the Supplementary Information.
