# Supplementary material for: High-throughput discovery of MHC class I- and II-restricted T cell epitopes using synthetic cellular circuits
Source: Nat Biotechnol. 2024 Jul 2;43(4):623–34. doi: 10.1038/s41587-024-02248-6 (PMC11994455; doi:10.1038/s41587-024-02248-6)
Supplement: Supplementary file 2 — MTA template. [file 41587_2024_2248_MOESM2_ESM.pdf]

Note: this letter may contain additional binding terms see page 2
For the complete terms of the UBMTA please see:
https://autm.net/AUTM/media/Surveys -Tools/UBMTAMaster-Agreement-v5.pdf

The Brigham and Women's Hospital UBMTA Implementing Letter

The purpose of this letter is to provide a record of the biological material transfer, to memorialize the agreement between the PROVIDER SCIENTIST (identified below) and the RECIPIENT SCIENTIST (identified below) to abide by all terms and conditions of the Uniform Biological Material Transfer Agreement ("UBMTA") March 8, 1995, and to certify that the RECIPIENT (identified below) organization has accepted and signed an unmodified copy of the UBMTA. The RECIPIENT organization's Authorized Official also will sign this letter if the RECIPIENT SCIENTIST is not authorized to certify on behalf of the RECIPIENT organization. The RECIPIENT SCIENTIST (and the Authorized Official of RECIPIENT, if necessary) should sign both copies of this letter and return one signed copy to the PROVIDER. The PROVIDER SCIENTIST may forward the material to the RECIPIENT SCIENTIST upon receipt of the signed copy from the RECIPIENT organization.

Please fill in all of the blank lines below:

1. PROVIDER: Organization providing the ORIGINAL MATERIAL:

   Organization: The Brigham and Women's Hospital, Inc.

   Address: 75 Francis Street
   Boston, MA 02115

2. RECIPIENT: Organization receiving the ORIGINAL MATERIAL:

   Organization: ____________________________

   Address: _________________________________

3. ORIGINAL MATERIAL (Enter description):

Human peptidome library, annotiatons, and associated analytic tools. Mouse peptidome library, annotations, and associated analytic tools. Human virome library, annotations, and associated analytic tools. Cell lines pertaining to Kohlgruber et al., Nature Biotechnology, 2024 paper associated with TCR-MAP antigen discovery.

4. Termination date for this letter (optional): ____________________

5. Transmittal Fee to reimburse the PROVIDER for preparation and distribution costs (optional).
   Amount: actual cost of shipping and handling.

Additional Binding Terms:
In the event of any conflict between the terms of the UBMTA and any additional binding terms set forth herein, such additional binding terms shall govern.

RECIPIENT shall only use the ORIGINAL MATERIAL for the noncommercial research purposes as set forth in Appendix A.

Each party further agrees not to use the name, trademark, service mark, logo, or other identifying characteristic ("Name") of the other party or any of its affiliates or any of their respective trustees, directors, officers, staff members, employees, students or agents in any advertising, promotional or sales literature, publicity or in any document employed to obtain funds or financing without the prior written approval of the party or individual whose Name is to be used, in the case of PROVIDER such approval to be given solely by the Public Affairs Department. This provision shall survive termination of this Agreement.

This Implementing Letter is effective when signed by all parties. The parties executing this Implementing Letter certify that their respective organizations have accepted and signed an unmodified copy of the UBMTA with additional binding terms above, if any, and further agree to be bound by its terms, for the transfer specified above.

If RECIPIENT ORGANIZATION is not a signatory of the UBMTA Master Agreement published in the Federal Register on March 8, 1995, RECIPIENT ORGANIZATION agrees to be bound by the terms of the UBMTA with additional binding terms above, if any, for the purposes of this transaction only.

PROVIDER SCIENTIST certifies having read and understood the entire agreement:

Name: Dr. Stephen Elledge

Title: Professor/HHMI

Investigator Address: 77 Avenue Louis

Pasteur, NRB 160, Boston MA 02115

Signature: Steph Ell

Date: ________________________

RECIPIENT SCIENTIST certifies having read and understood the entire agreement:

Name: ________________________________

Title: _________________________________

Address: ______________________________

Signature: _____________________________
Date: _________________________________

RECIPIENT ORGANIZATION CERTIFICATION

Authorized Official: ____________________________

Title: ____

Address:_______________________________________

Signature: _____________________________
Date: _________________________________

PROVIDER ORGANIZATION CERTIFICATION

Authorized Official:

Title:

Address: Mass General Brigham Innovation
Transactional Affairs Group
399 Revolution Drive, 7th Floor
Somerville, MA 02145

Signature:

Date:

APPENDIX A

Research Plan:
